# PnMYB4 negatively modulates saponin biosynthesis in *Panax notoginseng* through interplay with PnMYB1

**DOI:** 10.1093/hr/uhad134

**Published:** 2023-07-05

**Authors:** Jinhui Man, Yue Shi, Yuying Huang, Xiaoqin Zhang, Xin Wang, Shanhu Liu, Gaojie He, Kelu An, Dongran Han, Xiaohui Wang, Shengli Wei

**Affiliations:** School of Chinese Materia Medica, Beijing University of Chinese Medicine, Beijing 102488, China; School of Life and Science, Beijing University of Chinese Medicine, Beijing 102488, China; School of Chinese Materia Medica, Beijing University of Chinese Medicine, Beijing 102488, China; School of Chinese Materia Medica, Beijing University of Chinese Medicine, Beijing 102488, China; School of Chinese Materia Medica, Beijing University of Chinese Medicine, Beijing 102488, China; School of Chinese Materia Medica, Beijing University of Chinese Medicine, Beijing 102488, China; School of Chinese Materia Medica, Beijing University of Chinese Medicine, Beijing 102488, China; School of Chinese Materia Medica, Beijing University of Chinese Medicine, Beijing 102488, China; School of Life and Science, Beijing University of Chinese Medicine, Beijing 102488, China; Modern Research Center for Traditional Chinese Medicine, Beijing Institute of Traditional Chinese Medicine, Beijing University of Chinese Medicine, Beijing 102488, China; School of Chinese Materia Medica, Beijing University of Chinese Medicine, Beijing 102488, China

## Abstract

Saponins are the main triterpenoid ingredients from *Panax notoginseng*, a well-known Chinese medicine, and are important sources for producing drugs to prevent and treat cerebrovascular and cardiovascular diseases. However, the transcriptional regulatory network of saponin biosynthesis in *P. notoginseng* is largely unknown. In the present study we demonstrated that one R2R3-MYB transcription factor, designated *PnMYB4*, acts as a repressor of saponin accumulation. Suppression of *PnMYB4* in *P. notoginseng* calli significantly increased the saponin content and the expression level of saponin biosynthetic genes. PnMYB4 directly bound to the promoters of key saponin biosynthetic genes, including *PnSS*, *PnSE*, and *PnDS*, to repress saponin accumulation. PnMYB4 and the activator PnMYB1 could interacted with PnbHLH, which is a positive regulator of saponin biosynthesis, to modulate the biosynthesis of saponin. PnMYB4 competed with PnMYB1 for binding to PnbHLH, repressing activation of the promoters of saponin structural genes induced by the PnMYB1-PnbHLH complex. Our study reveals that a complex regulatory module of saponin biosynthesis is associated with positive and negative MYB transcriptional regulators and provides a theoretical basis for improving the content of saponins and efficacy of *P. notoginseng*.

## Introduction

*Panax notoginseng* (Burk.) F.H. Chen, which is known as sanqi, tianqi, or sanchi, is a valuable and famous traditional Chinese medicine for hemostasis, hemoptysis, and hematoma [1]. Furthermore, *P. notoginseng* has been widely used to prevent and manage cerebrovascular and cardiovascular disease [2]. Saponins are the principal triterpenoid ingredients of *P. notoginseng*, and approximately 100 saponins have been identified from *P. notoginseng* [3]. Pharmacological investigation showed that saponins have numerous functions, including cerebral vasodilation, blood pressure regulation, and hypoglycemic, hemostasis, anti-inflammatory, antioxidation, antitumor, antiapoptotic, antiedema and neuronal protection activities [4]. The demand for *P. notoginseng* and saponins has increased significantly because of increased application. Although the synthesis of some saponins in yeast is feasible at present [5], the *P. notoginseng* plant remains the principal natural source of saponins. However, the long growth cycle, continuous cropping obstacles, and harsh growth environment limit the production of *P. notoginseng* and saponins. Therefore, understanding the regulatory mechanism that modulates saponin biosynthesis will provide an efficient means for the production of saponins with important economic and commercial value and the cultivation of high-quality and high-yielding *P. notoginseng*.

The saponin biosynthetic pathway has been well characterized. Generally, the first committed step, the mevalonic acid (MVA) pathway and the methylerythritol phosphate (MEP) pathway result in the production of farnesyl diphosphate (FPP) and geranyl diphosphate [6]. This is followed by the conversion of two FPPs into the isoprenoid squalene catalyzed by squalene synthase (SS). The first oxygenation step of saponin biosynthetic pathway is catalyzed by squalene epoxidase (SE), which generates the epoxidation of the double bond of squalene to produce 2,3-oxidosqualene. Subsequently, dammarenediol-II synthase (DS) catalyzes the cyclization of 2,3-xidosqualene to generate dammarenediol. Further glycosylation, oxidation, and hydroxylation reactions are catalyzed by different glycosyltransferases (GT) and cytochrome P450 monooxyenases (CYP450), finally forming various saponins with various structures [7]. Moreover, overexpression of *PnSS*, *PnSE*, and *PnDS* significantly increased the accumulation of saponins in calli, suggesting that *PnSS*, *PnSE*, and *PnDS* are the key biosynthetic genes for saponin biosynthesis [8]. Compared with increasing information on saponin biosynthetic genes, the regulatory network controlling saponin biosynthesis remains to be further verified.

Terpenoid biosynthetic genes are always modulated by members of various transcription factor (TF) families, such as MYB, bHLH, ARF, WRKY, and NAC [9]. Among these TFs, R2R3-MYB TFs with an R2R3-type MYB domain have emerged as the major regulators in the terpenoid biosynthesis pathway [9]. For example, AtMYB21 [9] and AtMYB24 [10] can positively regulate the accumulation of sesquiterpenes, PtMYB14 can induce the synthesis of volatile terpenoids by regulating the MVA pathway [11], and Reduced Carotenoid Pigmentation (RCP1), which is an R2R3-MYB from *Erythranthe (Mimulus lewisii*), serves as the positive regulator of carotenoid levels in flowers [12]. In contrast to MYB activators, which are associated with the modulation of terpenoid accumulation, investigation of the MYB repressors has attracted increasing interest. In *Artemisia annua*, the R2R3-MYB TF TLR1 negatively modulates the accumulation of artemisinin and trichome density [13]. *SIMYB75* negatively modulates the formation of sesquiterpenes through specific combination of the *SITPS12*, *SITPS31*, and *SITPS3* promoters [14]. However, hierarchical interaction between the R2R3-MYB activators and repressors involved in terpenoid accumulation remains elusive.

Recent reports have demonstrated that the biosynthesis of terpenoids might be modulated by a synergistic and hierarchical regulatory network organized by various TFs. MYB21 has been shown to interact with MYC2 to generate an MYB-bHLH complex to modulate sesquiterpene accumulation in *Arabidopsis thaliana* and *Freesia hybrida* [9]. In *A. annua*, AabHLH112 specially combined with the promoter of *AaERF1* [15], which could then bind to the promoters of artemisinin structural genes and positively modulate the accumulation of artemisinin [16]. In *Catharanthus roseus*, the terpenoid indole alkaloid (TIA) pathway is modulated by CrMYC2 (the bHLH TF) regulating *ORCA2* and *ORCA3*, which in turn control the expression of a subset of alkaloid biosynthetic genes [17]. Compared with the synergistic TFs participating in the other terpenoid metabolism processes, such as sesquiterpene formation [18], artemisinin metabolism [19], and TIA synthesis [20], the interaction and hierarchical regulation of the TFs associated with the regulation of saponin accumulation remain elusive.

In *P. notoginseng*, the R2R3-MYB TF PnMYB1 [21] and PnbHLH [22] function as positive regulators of saponin accumulation. PnMYB3 can bind to the key saponin biosynthetic genes *PnSS* and *PnDS* [23]. Alternatively, no R2R3-MYB TFs have been characterized as negative regulators of the saponin biosynthetic pathway. We demonstrated that the new R2R3-MYB TF PnMYB4 acts as a repressor that regulates saponin biosynthesis in *P. notoginseng*. PnMYB4 and PnMYB1 interacted with PnbHLH to regulate the transcription of the key saponin biosynthetic genes *PnSS*, *PnSE*, and *PnDS*, and saponin biosynthesis. Furthermore, PnMYB4 competes with the activator PnMYB1 to bind to the PnbHLH protein, which suggests a regulatory network for saponin accumulation. These results enhance our understanding of the transcriptional modulation of saponin accumulation, and will pave the way for improving the content of saponin and efficacy of *P. notoginseng*.

## Results

### Identification of R2R3-MYB transcription regulators that potentially negatively regulate saponin biosynthesis

A previous report indicated that the saponin contents in the main roots were markedly higher than those of in the rootlets in *P. notoginseng* [38]. Our results also revealed that the contents of saponins, including R_1_, Rg_1_, and Rb_1_, in main roots of *P. notoginseng* were evidently higher than those in rootlets (Supplementary Data Fig. S1A and B). Transcriptome sequencing demonstrated that a total of 99 225 and 106 499 genes were analyzed in the main roots and rootlets, respectively. The number of differentially expressed genes (DEGs) between the main roots and rootlets was 14 836, and the expression levels of 9966 DEGs in the main roots were higher than those in the rootlets, while the transcript levels of 4870 DEGs in the main roots were lower than those in the rootlets (Supplementary Data Fig. S1C). Comparative transcriptome analysis indicated that in the main roots the expression level of genes including *PnACAT*, *PnHMGCS*, *PnHMGCR*, *PnMVK*, *PnPMK*, *PnMDD*, *PnDXS*, *PnDXR*, *PnispD*, *PnispE*, *PnispH*, *PnispG*, *PnIDI*, *PnGGPS*, *PnFDPS*, *PnSS*, *PnSE*, and *PnDS*, which were associated with the biosynthesis of saponins, were higher than those in rootlets, consistent with the results of saponin content analysis in main roots and rootlets (Supplementary Data Fig. S1D). Given that R2R3-MYB TFs could transcriptionally modulate terpenoid production, we analyzed the differentially expressed R2R3-MYB genes between the main roots and the rootlets. The results indicated that there were 14 differentially expressed MYB genes, the expression level of four *MYB* genes being higher in the main roots than in the rootlets; the transcript levels of 10 *MYB*s were lower in the main roots than those in the rootlets (Supplementary Data Fig. S1E). To further verify the reliability of DEGs between the main roots and rootlets, the abundances of saponin biosynthetic genes and *MYB* genes were detected with qRT–PCR. The transcript levels of *PnACAT*, *PnHMGCS*, *PnMVK*, *PnMDD*, *PnispE*, *PnispH*, *PnispG*, *PnGGPS*, *PnFDPS*, *PnSS*, *PnSE*, and *PnDS* in the main roots were remarkably higher than those in the rootlets (Supplementary Data Fig. S1F), consistent with the comparative transcriptome analysis. The abundance of eight *MYB* genes in the main roots were markedly lower than those in the rootlets, while the expression level of *PnMYB4* indicated the greatest difference between the main roots and rootlets (Supplementary Data Fig. S1G), suggesting that *PnMYB4* might be associated with the negative regulation of saponin biosynthesis.

### Isolation and characterization of PnMYB4 from *P. notoginseng*

**Figure 1:**
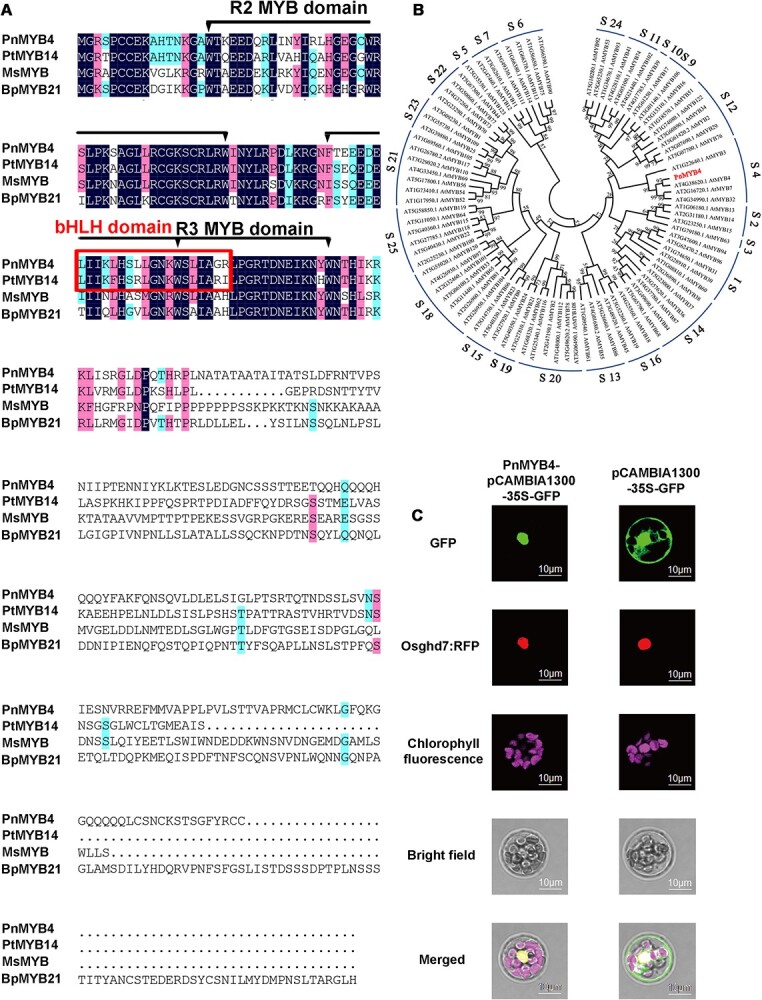
Sequence analysis of PnMYB4 and subcellular localization of PnMYB4. (A) Multiple sequence alignment of PnMYB4 protein from *P. notoginseng* with known triterpenoid-specific R2R3-MYB regulators from other plants. The alignment was performed with the DNAMAN program. The R2R3 conservative structure and conserved bHLH-binding motif are marked, respectively. Pn, *Panax notoginseng*; Pt, *Pinus taeda*; Ms, *Mentha spicata*; Bp, *Betula platyphylla*. (B) Phylogenetic analysis of PnMYB4 and R2R3-MYB proteins from *A. thaliana*, generated by MEGA11. Bootstrap values are indicated with numbers on each branch. (C) Subcellular location of PnMYB4 in *A. thaliana* protoplasts. GFP and RFP fluorescence signals were captured with a confocal microscope 18 h after transformation. Scale bars = 10 μm.

The *PnMYB4* cDNA sequence was successfully cloned through heterologous screening of transcriptome data of *P. notoginseng*. The full-length *PnMYB4* (GenBank: ON783667) contained an ORF of 792 bp encoding a protein of 263 amino acid residues with a predicted molecular mass (MW) of ~30.4 kDa. To further determine the sequence features, PnMYB4 was aligned and compared with PtMYB14, MsMYB, and BpMYB21, which play important roles in triterpenoid formation. DNAMAN analysis results showed that PnMYB4 contained both the conserved R2 and R3 structures and the conserved bHLH-interacting motif (D/E)LX_2_(R/K)X_3_LX_6_LX_3_R, suggesting that PnMYB4 is the R2R3-MYB TF and putatively interacts with bHLH TF (Fig. 1A). Furthermore, the sequence of PnMYB4 was compared with the *Arabidopsis thaliana* R2R3-MYB TFs to generate a phylogenetic tree. PnMYB4 was phylogenetically close to AtMYB4 (Fig. 1B), which negatively regulates anthocyanin or phenylpropanoid biosynthesis, suggesting that PnMYB4 is the candidate regulation repressor.

We verified the subcellular localization of PnMYB4 by co-transforming the PnMYB4:GFP fusion construct and nuclear marker Osghd7:RFP in the mesophyll protoplast of *A. thaliana*. The green fluorescence of PnMYB4:GFP fusion protein exclusively accumulated in the nucleus and strictly colocalized with Osghd7:RFP (Fig. 1C), suggesting that PnMYB4 was a nuclear-localized protein.

### RNAi-mediated suppression and CRISPR/Cas9-mediated targeted mutagenesis of *PnMYB4* induced saponin biosynthetic gene expression and saponin accumulation in *P. notoginseng* calli

**Figure 2:**
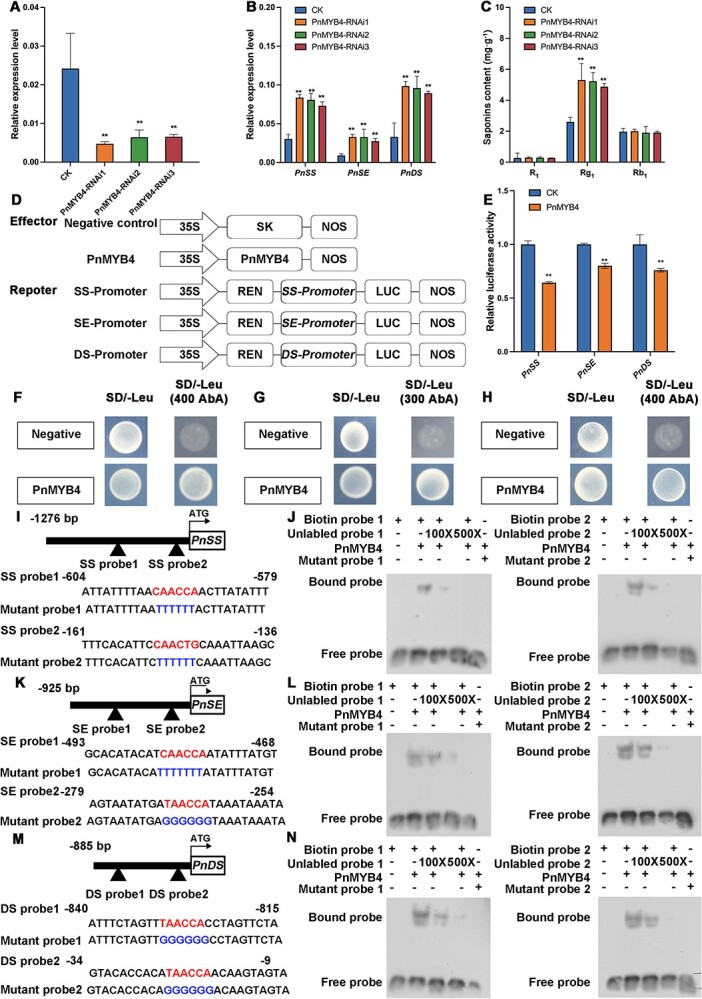
PnMYB4 inhibits *PnSS*, *PnSE*, and *PnDS* expression, represses saponin accumulation in *P. notoginseng* calli, and binds to the promoters of *PnSS*, *PnSE*, and *PnDS*. (A) Relative expression level of *PnMYB4* in control (CK) and PnMYB4-RNAi transgenic calli. (B) Transcript level of saponin biosynthetic genes, including *PnSS*, *PnSE*, and *PnDS* in CK and PnMYB4-RNAi transgenic calli. (C) Accumulation of R_1_, Rg_1_, and Rb_1_ in CK and *PnMYB4* -RNAi transgenic calli. Relative expression levels of *PnMYB4*, *PnSS*, *PnSE*, and *PnDS* were quantified by qRT–PCR and normalized with the amount of *PnACT2*. Asterisks indicate statistically significant differences relative to CK. Statistical analysis was performed by one-way ANOVA with Duncan’s multiple range tests to separate means. ^**^P < .01, ^*^P < .05. (D) Schematic diagrams of effector and reporter vectors used in dual-LUC assays. (E) Dual-LUC reporter assays showing that the transcription activity of *PnSS*, *PnSE*, and *PnDS* promoters was repressed by PnMYB4. Asterisks indicate significant differences (^**^P < .01). Data are the mean ± standard error of six biological replicates. (F–H) Y1H assays indicating the binding of PnMYB4 to the promoters of *PnSS* (F), *PnSE* (G), and *PnDS* (H). The negative control was pGADT7. (I, K, M) Sequence of probes from *PnSS* (I), *PnSE* (K), and *PnDS* (M) promoter fragments. (J, L, N) EMSA indicating the specific combination between PnMYB4 and MBSs in the promoters of *PnSS* (J), *PnSE* (L), and *PnDS* (N). Unlabeled probes with the same sequence as the biotin-labeled probes and mutant probes with TTTTTT and GGGGGG, which mutated from CAACC(T)A(G) and TAACCA, were used as competitors.

*P. notoginseng* is a perennial plant and there are various endophytic fungi and bacteria in roots, stems, leaves, and flowers. These make studies using fresh plants and establishing calli from roots, stems, leaves, and flowers difficult and inconvenient. Thus, *P. notoginseng* calli were induced from sprouts and the content of saponins in the calli was carefully analyzed by UPLC (Supplementary Data Fig. S2A and B). The results revealed that the calli induced from sprouts also contain saponins, and the content of Rg_1_ was most abundant, followed by Rb_1_ and then R_1_, similar to the accumulation pattern of saponins in the main root and rootlets (Supplementary Data Fig. S2B). To verify the function of PnMYB4 in the regulation of saponin biosynthesis, the expression of *PnMYB4* in calli was suppressed by the RNAi method, the expression level of saponin biosynthetic genes was detected by qRT–PCR, and the content of saponins, including R_1_, Rg_1_, and Rb_1_, was analyzed by UPLC. In the RNAi transgenic calli, the transcript level of *PnMYB4* was markedly decreased (Fig. 2A), whilst expression levels of *PnSS*, *PnDS*, and *PnSE* were significantly increased (Fig. 2B), and the content of Rg_1_ in *PnMYB4* RNAi transgenic calli was significantly increased (Fig. 2C). To further modify the function of *PnMYB4*, we used the CRISPR/Cas9 system to precisely edit the *PnMYB4* gene in calli (Supplementary Data Fig. S3A), and then analyzed the expression levels of saponin biosynthetic genes and the content of saponins. The results indicated that the transcript levels of *PnSS*, *PnDS*, and *PnSE* were significantly increased, and the contents of Rg_1_ and Rb_1_ were also markedly increased (Supplementary Data Fig. S3B and C). The above results suggested that *PnMYB4* could negatively regulate saponin biosynthesis through regulating expression of *PnSS*, *PnSE*, and *PnDS*.

### PnMYB4 binds to the promoters of *PnSS*, *PnSE*, and *PnDS* and represses their transcription activity

*PnSS*, *PnSE*, and *PnDS* were the key saponin biosynthetic genes, and downregulated PnMYB4 could significantly increase the expression of these genes. Furthermore, there were two putative different MYB binding sites (MBSs) [(C/T)AAC(T/C)(G/A)] within the 1276-bp promoter fragment of *PnSS* and the 925-bp promoter of *PnSE*, respectively, while the 885-bp *PnDS* promoter contained two identical MBSs (TAACCA) (Supplementary Data Tables S2 andS3), indicating that PnMYB4 may bind to the promoters of *PnSS*, *PnSE*, and *PnDS*. The dual-LUC assays demonstrated that PnMYB4 was able to markedly inhibit the transcription activity from the promoters of *PnSS*, *PnSE*, and *PnDS* (Fig. 2D and E), indicating that PnMYB4 is a repressor of saponin biosynthesis. We then carried out the yeast one-hybrid (Y1H) assay, which demonstrated that PnMYB4 could bind to the promoters of *PnSS*, *PnSE*, and *PnDS* (Fig. 2F and H). To identify the binding sites for PnMYB4 in the promoters of *PnSS*, *PnSE*, and *PnDS*, electrophoretic mobility shift assays (EMSAs) were performed. The promoter fragments that contained the MBSs for the promoters of *PnSS*, *PnSE*, and *PnDS* were amplified and used as probes for EMSA (Fig. 2I, K, and M). The purified recombinant PnMYB4 protein bound to the promoter fragments of *PnSS*, *PnSE*, and *PnDS*, causing mobility shifts; additionally, the binding of labeled fragments of *PnSS*, *PnSE*, and *PnDS* by PnMYB4 could be abrogated by increasing concentrations (100× and 500× excess) of unlabeled competitor probes (Fig. 2J, L, and N), and the mutant probes of *PnSS*, *PnSE*, and *PnDS* by PnMYB4 could not be bound, indicating that PnMYB4 could bind the two specific MBSs in the *PnSS*, *PnSE*, and *PnDS* promoters.

### PnMYB4 interacts with PnbHLH to regulate saponin biosynthesis

**Figure 3:**
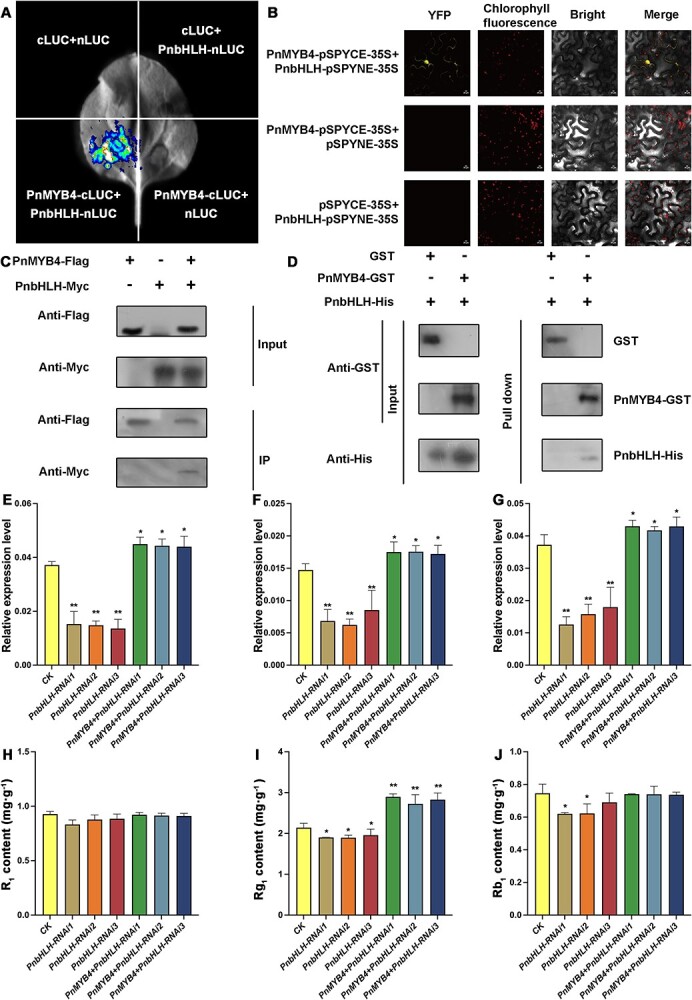
PnMYB4 interacts with PnbHLH protein to regulate saponin biosynthesis. (A) Interaction between PnMYB4 and PnbHLH in LCI assays. PnMYB4-cLUC+PnbHLH-nLUC with other negative controls containing PnMYB4-cLUC+nLUC, cLUC+PnbHLH-nLUC, and cLUC +nLUC were infiltrated into different parts of the same *N. benthamiana* leaf. (B) Interaction between PnMYB4 and PnbHLH analyzed with BiFC assays. YFP signals were detected by confocal microscopy 48 h after infiltration. Scale bars = 20 μm. (C) Co-IP assay to verify the interaction between PnMYB4 and PnbHLH in *N. benthamiana*. *N.benthamiana* leaves infiltrated with different combinations, shown above the panels, were harvested at 48 h. Total proteins of leaves were immunoprecipitated with anti-Flag beads. Immunoprecipitated and total proteins were detected by western blotting with anti-Flag and anti-Myc antibodies. (D) GST pull-down analyses of interaction between PnMYB4 and PnbHLH *in vitro*. GST-tagged PnMYB4 was incubated with PnbHLH-His. Input and pull-down proteins were analyzed by western blotting analysis using anti-GST or anti-His antibodies. (E) Transcript level of *PnSS* in control (CK), *PnbHLH*-RNAi, and *PnMYB4* + *PnbHLH*-RNAi transgenic calli. (F) Transcript level of *PnSE* in CK, *PnbHLH*-RNAi, and *PnMYB4* + *PnbHLH*-RNAi transgenic calli. (G) Transcript level of *PnDS* in CK, *PnbHLH*-RNAi, and *PnMYB4* + *PnbHLH*-RNAi transgenic calli. (H) Accumulation of R_1_ in CK, *PnbHLH*-RNAi, and *PnMYB4* + *PnbHLH*-RNAi transgenic calli. (I) Accumulation of Rg_1_ in CK, *PnbHLH*-RNAi, and *PnMYB4* + *PnbHLH*-RNAi transgenic calli. (J) Accumulation of Rb_1_ in CK, *PnbHLH*-RNAi, and *PnMYB4* + *PnbHLH*-RNAi transgenic calli. Relative expression levels of *PnSS*, *PnSE*, and *PnDS* were quantified by qRT–PCR and normalized with the amount of *PnACT2*. Asterisks show statistically significant differences relative to CK. Statistical analysis was performed by one-way ANOVA with Duncan’s multiple range tests to separate means. ^**^P < .01, ^*^P < .05.

A previous investigation showed that PnbHLH is an activator of saponin biosynthesis and could activate the transcription of *PnSS*, *PnSE*, and *PnDS* [22]. The above results also indicated that PnMYB4 contains a conserved bHLH-interacting motif (Fig. 1A). In light of these findings, we hypothesized that PnMYB4 may interact with PnbHLH. We firstly performed a luciferase complementation imaging (LCI) assay to demonstrate the interaction of PnMYB4 and PnbHLH in *Nicotiana benthamiana*. In transient expression assays, the leaves co-transformed with PnMYB4-cLUC and PnbHLH-nLUC recombinant constructs emitted LUC signals, indicating the interaction between PnMYB4 and PnbHLH, while no luminescence signals were detected in the leaves co-transformed with other combinations of plasmids, including nLUC+cLUC, cLUC+PnbHLH-nLUC, and PnMYB4-cLUC+nLUC (Fig. 3A). To demonstrate where PnMYB4 and PnbHLH interact in cells, the bimolecular fluorescent complementation (BiFC) assay was carried out. Reconstitution of YFP signals was captured in the nucleus of epidermal cells co-transformed with the recombinant plasmids PnMYB4-pSPYCE and PnbHLH-pSPYNE (Fig. 3B). However, no yellow fluorescence was detected in epidermal cells co-infiltrated with PnMYB4-pSPYCE and empty pSPYNE, or PnbHLH-pSPYNE and pSPYCE (Fig. 3B). To further validate whether PnMYB4 could form a complex with PnbHLH, a co-immunoprecipitation (Co-IP) assay was performed using the *N. benthamiana* transient expression system. *Agrobacterium tumefaciens* harboring recombinant plasmids expressing PnMYB4-Flag and PnbHLH-Myc were infiltrated into *N. benthamiana* leaf epidermal cells. Total proteins from the infiltrated leaves were precipitated by anti-Flag affinity beads and western blotted with anti-Flag and anti-Myc antibodies. As shown in Fig. 3C, PnMYB4-Flag specifically co-immunoprecipitated with PnbHLH-Myc. We next examined the direct interaction between PnMYB4 and PnbHLH using GST pull-down assays. The results indicated that PnbHLH-His was directly precipitated with PnMYB4-GST *in vivo*, but not with GST (Fig. 3D). These results suggested that PnMYB4 physically interacts with PnbHLH *in vivo* and *in vitro*.

To understand the regulatory effects of PnbHLH and PnMYB4 on the biosynthesis of saponins, dual-LUC assays were carried out. Infiltration of leaves with PnbHLH resulted in a marked rise in the transcription activity from the *PnSS*, *PnSS*, and *PnDS* promoters (Supplementary Data Fig. S4A–C). However, when PnbHLH was co-infiltrated with PnMYB4, the transcriptional activation of the *PnSS*, *PnSE*, and *PnSE* promoters was markedly repressed compared with that of PnbHLH alone (Supplementary Data Fig. S4A–C). After knockdown of the expression level of *PnbHLH* using the RNAi method (Supplementary Data Fig. S4D), the abundances of *PnSS*, *PnSE*, and *PnDS* and the accumulations of Rg_1_, Rb_1_, and R_1_ were significantly decreased (Fig. 3E–J). When the expression of *PnMYB4* and PnbHLH was inhibited with the RNAi method (Supplementary Data Fig. S4E and F), the abundances of *PnSS*, *PnSE*, and *PnDS* were significantly decreased compared with the control calli, but were upregulated compared with those of in PnbHLH RNAi transgenic calli (Fig. 3E–G). Furthermore, the content of Rg_1_ was increased in the calli in which the expression of PnbHLH and PnMYB4 were downregulated (Fig. 3H–J). These results suggested that PnMYB4 inhibited the transcriptional activation function of PnbHLH.

### Transcriptional activator PnMYB1 interacts with PnbHLH to regulate saponin biosynthesis

**Figure 4:**
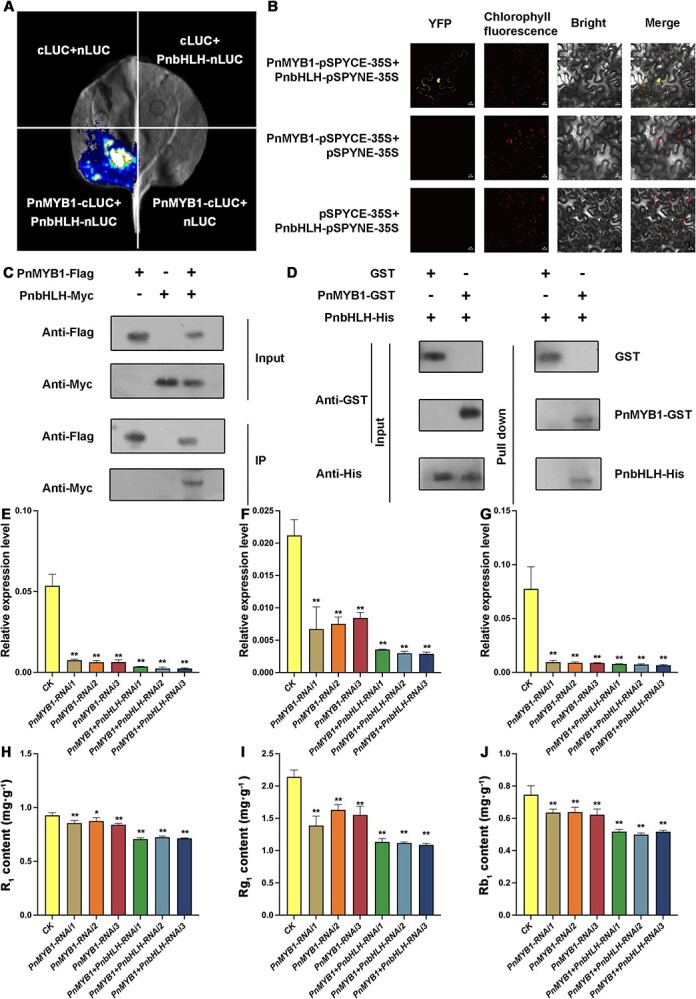
PnMYB1 interacts with PnbHLH to promote saponin accumulation. (A) LCI assay to analyze the interaction between PnMYB1 with PnbHLH. Luminescence of *N. benthamiana* was visualized 36–48 h after agroinfiltration. (B) BiFC analysis of interaction between PnMYB1 and PnbHLH in *N. benthamiana* leaves. YFP signals were captured by confocal microscopy at 48 h. Scale bars = 20 μm. (C) Co-IP analysis of interaction between PnMYB1 and PnbHLH. PnMYB1-Flag protein and PnbHLH-Myc were co-expressed in *N. benthamiana* leaves and detected at 48 h. Total proteins from infiltrated leaves were immunoprecipitated with anti-Flag beads. Total proteins and immunoprecipitated proteins were analyzed by western blotting using anti-Flag and anti-Mys antibodies. (D) GST pull-down assay to reveal in *vitro* interaction between PnMYB1 with PnbHLH. Purified PnMYB1-GST was incubated with PnbHLH-His. Input and pull-down proteins were analyzed by western blotting using anti-GST or anti-His antibodies. (E) Transcript level of *PnSS* in control (CK), *PnMYB1*-RNAi, and *PnMYB1* + *PnbHLH*-RNAi transgenic calli. (F) Transcript level of *PnSE* in CK, *PnMYB1*-RNAi, and *PnMYB1* + *PnbHLH*-RNAi transgenic calli. (G) Transcript level of *PnDS* in CK, *PnMYB1*-RNAi, and *PnMYB1* + *PnbHLH*-RNAi transgenic calli. (H) Accumulation of R_1_ in CK, *PnMYB1*-RNAi, and *PnMYB1* + *PnbHLH*-RNAi transgenic calli. (I) Accumulation of Rg_1_ in CK, *PnMYB1*-RNAi, and *PnMYB1* + *PnbHLH*-RNAi transgenic calli. (J) Accumulation of Rb_1_ in CK, *PnMYB1*-RNAi, and *PnMYB1* + *PnbHLH*-RNAi transgenic calli. Relative expression levels of *PnSS*, *PnSE*, and *PnDS* were quantified by qRT–PCR and normalized with the amount of *PnACT2*. Asterisks reveal statistically significant differences relative to CK. Statistical analysis was performed by one-way ANOVA with Duncan’s multiple range tests to separate means. ^**^P < .01, ^*^P < .05.

**Figure 5:**
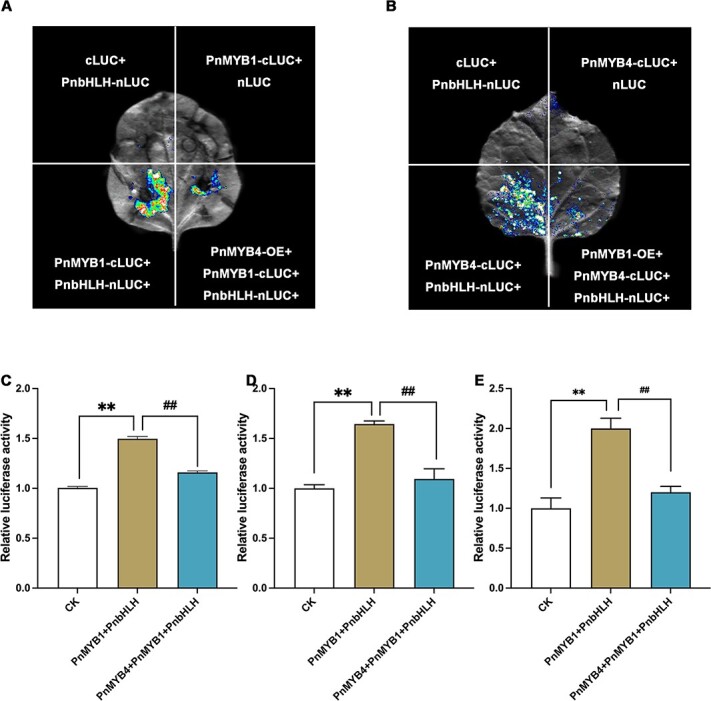
PnMYB4 regulates saponin biosynthetic gene transcription by interfering with the activation capacity of the PnMYB1-PnbHLH complex. (A) LCI competitive assays indicated that PnMYB4 reduced the affinities of the PnMYB1-PnbHLH complex. (B) LCI competitive assays indicated that PnMYB1 reduced the affinities of the PnMYB4-PnbHLH complex. (C–E) Dual-LUC assays showed that PnMYB4 markedly decreased the transcription rate from the promoters of *PnSS* (C), *PnSE* (D), and *PnDS* (E) relative to that induced by PnMYB1-PnbHLH complex in *N. benthamiana* leaf cells. Statistical analysis was performed by one-way ANOVA with Duncan’s multiple range tests to separate means. Asterisks indicate significant differences. ^**^P < .01, ^*^P < .05, ^##^*P* < .01, ^#^*P* < .05.

Previous reports showed that *PnMYB1* positively regulated the biosynthesis of saponins by promoting *PnSE* and *PnDS* [21]. Our results also revealed that the abundance of *PnMYB1* in the main root was markedly higher than that in the rootlets (Supplementary Data Fig. S5A and B). We cloned the *PnMYB1* sequence from *P. notoginseng* and performed amino acid analysis. The results revealed that PnMYB1 contained the conserved bHLH-interacting motif (Supplementary Data Fig. S6), suggesting that PnMYB1 might also interact with the PnbHLH protein. Therefore, LCI, BiFC, Co-IP, and GST-pull down assays were carried out to test the interaction between PnMYB1 and PnbHLH. As expected, the LCI assay showed that co-expression of PnMYB1-cLUC and PnbHLH-nLUC resulted in reconstitution of LUC signals, while PnMYB1-cLUC/PnbHLH-nLUC with other negative controls failed to generate luminescence (Fig. 4A). BiFC analysis showed that coinfiltration of PnMYB1-pSPYCE and PnbHLH-pSPYNE constructs reconstituted YFP fluorescence in the nuclei of *N. benthamiana* leaf cells (Fig. 4B). Co-IP assays showed that PnbHLH-Myc, rather than Myc, was precipitated with PnMYB1-Flag (Fig. 4C). GST pull-down assays demonstrated that the PnbHLH-His protein was precipitated with PnMYB1-GST, but not with GST (Fig. 4D). Collectively, PnMYB1 interacts directly with PnbHLH *in vivo* and *in vitro*. Furthermore, the dual-LUC assays revealed that PnMYB1 could not only activate transcription from the *PnSS* and *PnSE* promoters, consistent with previous reports, but also induce transcription from the *PnDS* promoter (Supplementary Data Fig. S7A–C). Co-expression with the saponin biosynthesis activator PnMYB1 and PnbHLH led to a significant rise in transcription from the *PnSS*, *PnSE*, and *PnDS* promoters (Fig. 5C–E). When the expression of *PnMYB1* was downregulated with the RNAi method (Supplementary Data Fig. S7D), the expression levels of *PnSS*, *PnSE*, and *PnDS* and the accumulations of Rg_1_, Rb_1_, and R_1_ were significantly decreased (Fig. 4E–J). Moreover, when the expression levels of *PnMYB1* and *PnbHLH* were simultaneously downregulated using the RNAi method (Supplementary Data Fig. S7E and F), the expression levels of *PnSS*, *PnSE*, and *PnDS* and the accumulations of Rg_1_, Rb_1_, and R_1_ were significantly decreased compared with those in the calli which there was downregulation of the expression of *PnMYB1* alone, or the control calli (Fig. 4E–J). These results indicated that PnMYB1 could interact with PnbHLH to positively regulate saponin biosynthesis.

### PnMYB4 competes with PnMYB1 to interact with PnbHLH and regulate the transcription of *PnSS*, *PnSE*, and *PnDS*

A competitive LCI assay was used to reveal the competition between PnMYB1 and PnMYB4 for the interaction with PnbHLH. PnMYB1-cLUC and PnbHLH-nLUC were co-infiltrated with or without PnMYB4. The results indicated that co-infiltration of PnMYB1–cLUC and PnbHLH–nLUC produced strong luminescence, while the concomitant transient expression of PnMYB4 significantly decreased signal intensity (Fig. 5A). The same assays were performed using PnMYB4-cLUC and PnbHLH-nLUC with or without PnMYB1. PnMYB1 decreased the PnMYB4-cLUC and PnbHLH-nLUC interaction (Fig. 5B). Collectively, these results suggested that PnMYB4 competed with PnMYB1 for interaction with PnbHLH.

To further test the function of the coordinate action of PnMYB1 and PnMYB4 in regulation of saponin biosynthesis, we carried out dual-LUC assays. The results revealed that co-infiltration of leaves with PnMYB4 markedly decreased the transcription rate from the *PnSS*, *PnSE*, and *PnDS* promoters relative to that induced with the PnMYB1-PnbHLH complex (Fig. 5C–E). Furthermore, co-expression of PnMYB4, PnMYB1, and PnbHLH also led to a rise in transcription from the promoters of *PnSS*, *PnSE*, and *PnDS* (Fig. 5C–E). These results suggested that the interplay between PnMYB4 and PnMYB1 ultimately regulated saponin biosynthesis.

## Discussion

In recent years, saponin ingredients, which are triterpenoids isolated from *P. notoginseng* [3], have been used as medicines for the prevention and treatment of cardiovascular and cerebrovascular diseases [2]. A detailed understanding of the mechanism of saponin formation and regulation potentially provides a new avenue for improving the quality of *P. notoginseng* and identifying the drug lead compounds for new medicines. To date, most genes encoding the enzymes associated with saponin biosynthesis have been identified and characterized [8]. However, the regulatory mechanism responsible for saponin formation remains to be fully understood. In our study, we developed a regulatory network that involves both positive and negative R2R3-MYB regulators to modulate saponin biosynthesis.

**Figure 6:**
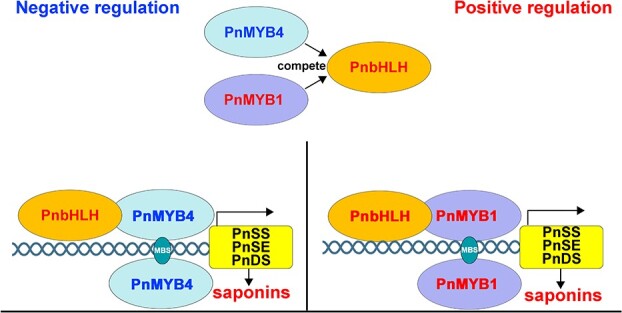
Working model of PnMYB4 in regulation of saponin biosynthesis in *P. notoginseng*. PnMYB4 could specifically combined with the promoters of the key saponin biosynthetic genes, including *PnSS*, *PnSE* and *PnDS*, and repress their transcription, resulting in inhibition of the accumulation of saponins in *P. notoginseng*. PnMYB1 could activate the expression of *PnSS*, *PnSE*, and *PnDS* and the accumulation of saponins. PnMYB4 and PnMYB1 could interact with the activator PnbHLH to regulate the production of saponins. PnMYB4 competes with PnMYB1 for binding to PnbHLH, ultimately regulating saponin biosynthesis.

### PnMYB4 is a repressor for saponin biosynthesis in *P. notoginseng*

R2R3-MYB TFs have been widely reported to participate in the modulation of plant secondary metabolism, including terpenoid biosynthesis [39]. PnMYB1 has been reported as a positive modulator of the biosynthesis of saponins in *P. notoginseng* [21]. Nevertheless, few R2R3-MYB family genes have been characterized to negatively regulate the saponin biosynthetic pathway. In our study, transcription sequencing and qRT–PCR analysis demonstrated that the expression level of *PnMYB4* in the main roots was significantly lower than in the rootlets. In contrast, the content of saponins in the main roots was markedly higher than that in the rootlets (Supplementary Data Fig. S1B). PnMYB4 was phylogenetically close to AtMYB4 (Fig. 1B), which is involved in negatively regulating the biosynthesis of anthocyanin or phenylpropanoid and the response to abiotic and biotic stresses [40, 41], suggesting PnMYB4 is a transcriptional repressor. Downregulation of *PnMYB4* in *P. notoginseng* calli using the RNAi method caused strong upregulation of the expression of *PnSS*, *PnSE*, and *PnDS*, and an enhanced accumulation of ginsenoside Rg_1_, which is the most abundant saponin in calli (Fig. 2A–C). These results revealed that PnMYB4 plays an important role in negatively regulating saponin biosynthesis.

R2R3-MYB TFs always specifically combine with the promoters of key biosynthetic genes to promote or repress the expression of biosynthetic genes and the production of terpenoid metabolites. For example, FhMYB21L2 showed high sequence homology with AtMYB21, could regulate *FhTPS1* expression by combining with the conserved MYB recognition sequences (MYBCORE box motif, CAACCG) in the *FhTPS1* promoter, and could modulate monoterpene accumulation [9]. In tomato, SlMYB75 directly interacted with the MYB core motif in the *SlTPS12*, *SlTPS31*, and *SlTPS35* promoters, and inhibited sesquiterpene saponin production in glandular trichomes [14]. *PnSS*, *PnSE*, and *PnDS* are the key genes for saponin biosynthesis, as shown by the finding that transgenic *P. notoginseng* calli overexpressing *PnSS*, *PnSE*, or *PnDS* significantly increased the accumulation of saponins. PlantCARE database analysis showed that the promoters of *PnSS*, *PnSE*, and *PnDS* all contain two putative MBSs [(C/T)AAC(T/C)(G/A)]. Our results demonstrated that PnMYB4 significantly inhibited transcription from the *PnSS*, *PnSE*, and *PnDS* promoters, and could specifically combine with two putative MBSs in the *PnSS*, *PnSE*, and *PnDS* promoter regions (Fig. 2D–N), suggesting that *PnSS*, *PnSE*, and *PnDS* are the target enzyme genes for PnMYB4 (Fig. 6). Functionally, PnMYB4 negatively regulates the biosynthesis of saponin through repressing the expression of *PnSS*, *PnSE*, and *PnDS.*

### The activator PnMYB1 and repressor PnMYB4 could interact with PnbHLH to modulate saponin accumulation

In many plant species, R2R3-MYB activators and bHLH TFs always form complexes to modulate plant secondary metabolism [42]. Accumulating evidence demonstrates that the conserved MYB-bHLH-WD40 (MBW) complex in higher plants modulates the expression level of biosynthetic genes to regulate flavonoid [43], proanthocyanin [44], and anthocyanin formation [45]. Compared with the MYB-bHLH complex participating in the modulation of anthocyanin, proanthocyanin, and flavonoid accumulation, a few studies have reported the function of the MYB-bHLH complex in terpenoid metabolism. Only one report demonstrated that, in *Freesia hybrida*, FhMYB21L2 activators could counteract the regulation of *FhTPS1* through interacting with FhbHLH, while AtMYB21 interacted with AtMYC2 to modulate *AtTPS11* expression and sesquiterpene production [9]. In addition to R2R3-MYB activators, the R2R3-MYB repressors, which regulate anthocyanin, proanthocyanin, and flavonoid biosynthesis, have been reported to interact with bHLH partners [46]. Some R2R3-MYB TFs, such as FaMYB1 [47] and MYB27 [48], do not directly combine with the promoters of biosynthetic genes but act as co-repressors that interact with bHLH partners or the MBW complex to repress structural gene expression, while other R2R3-MYB TFs, including PpMYB18 [49] and MaMYBPR [16], could bind to the promoters of target genes and interact with bHLH partners. However, few reports have indicated the interaction between R2R3-MYB repressors and bHLH TFs in regulation of terpenoid metabolism.

PnMYB1 and PnbHLH have been indicated to positively regulate the accumulation of saponins [22]. Sequence analysis indicated that PnMYB1 and PnMYB4 contain the conserved bHLH-interacting motif, suggesting that the activator PnMYB1 and repressor PnMYB4 could interact with PnbHLH. The interaction between PnbHLH and PnMYB1 or PnMYB4 *in vivo* or *in vitro* was confirmed with LCI, BiFC, Co-IP, and GST pull-down assays (Figs 3A–D and4A–D). The dual-LUC assays indicated that PnMYB4 could also significantly inhibit transcription from the *PnSS*, *PnSE*, and *PnDS* promoters induced by PnbHLH (Supplementary Data Fig. S4A–C), whilst PnMYB1 could markedly increase the transcriptional activation for the promoters of *PnSS*, *PnSE*, and *PnDS* induced by PnbHLH (Fig. 5C–E). Additionally, when the expression of *PnMYB4* and *PnbHLH* was downregulated using the RNAi method, the abundances of *PnSS*, *PnSE*, and *PnDS* and the accumulation of saponins were higher than those in the calli in which only *PnbHLH* was knocked down. However, when the expression of *PnMYB1* and *PnbHLH* was downregulated in calli, the expression levels of *PnSS*, *PnSE*, and *PnDS* and the content of saponins were lower than in the calli in which *PnbHLH* or *PnMYB1* was knocked down alone. Combining the results of biochemical data and genetic evidence, we confirmed for the first time that the repressor PnMYB4 and activator PnMYB1 could interact with PnbHLH to modulate the expression of saponin structural genes and saponin biosynthesis.

### Coordinated action between PnMYB4 and PnMYB1 plays an important role in modulation of saponin accumulation

Recent reports revealed that the coordinated actions of R2R3-MYB activators and repressors play vital roles in the modulation of biosynthesis of particular metabolites [49]. Some R2R3-MYB repressors that regulate anthocyanin, proanthocyanin, and flavonoid metabolism have been demonstrated to interact with bHLH partners to construct another competitive MBW complex that inhibits the expression of biosynthetic genes [46, 50]. In peach, the PpMYB18 repressor competed with PpMYB10, or PpMYBPA1 for binding to bHLH proteins to perform its passive repression function in the transcriptional regulation of anthocyanin and proanthocyanin biosynthesis [49]. MaMYBPRs may compete with the activator MaMYBPA for binding to bHLH cofactors, thus repressing the expression of proanthocyanin biosynthetic genes [33]. VvMYB14 and VvMYB30 function as the activator and repressor of stilbene accumulation, respectively, and VvMYB14 and VvMYB30 might cooperate with VvWRKY8 to form a ‘VvMYB14-VvWRKY8-VvMYB30’ module to balance stilbene biosynthesis [51]. In addition, coordinated actions between FaMYB10 and FaMYB44.2 finally results in the biosynthesis of sucrose in ripening strawberry fruits. Terpenoid biosynthesis may also be modulated by a transcription network associated with R2R3-MYB activators and repressors. Our results indicated that PnMYB1 and PnMYB4 are the activator and repressor of saponin biosynthesis, respectively, and PnMYB1 and PnMYB4 could interact with PnbHLH. Moreover, PnMYB4 competes with PnMYB1 to bind PnbHLH, and the transcriptional activation from *PnSS*, *PnSE*, and *PnDS* promoters induced by PnMYB1-PnbHLH were repressed by PnMYB4 (Fig. 6). These observations demonstrate that the interplay between PnMYB4 and PnMYB1 ultimately regulates the accumulation of saponin in *P. notoginseng*.

## Materials and methods

### Plant materials

Two-year-old *Panax notoginseng* main roots and rootlets were collected from Wenshan City, Yunan Province, China. The *P. notoginseng* seeds were stored in sand for about a month (30–40 days), and then the sprouts were cut into 3- to 4-cm pieces and surface-sterilized as described previously [24]. The treated sprouts were cultured in Murashige–Skoog (MS) medium containing 2 μg ml^−1^ dichlorophenoxyacetic acid (2,4-D), 1 μg ml^−1^ 6-benzylaminopurine (6-BA), 1 μg ml^−1^ kinetin (KT) and 2 μg ml^−1^ 1-naphthylacetic acid (NAA). After 1 month of incubation at 25°C in the dark, the calli were cultured on fresh MS medium.

### Analysis of content of saponins

Contents of *P. notoginseng* saponins, including notoginsenoside R_1_ (R_1_), ginsenoside Rg_1_ (Rg_1_) and ginsenoside Rb_1_ (Rb_1_) were determined using an ultra-performance liquid chromatography (UPLC) system on a Shimadzu ultra-high performance liquid chromatography system (SPD-M40, Japan) with a Waters Acquity UPLC^®^ BEH C18 reversed-phase column (Waters, USA, 2.1 mm × 100 mm, 1.7 μm) according to the method described in the Chinese Pharmacopeia [25] and by Jiang *et al*. [26]. Ten microliters of sample extract was detected via UPLC at 40°C. The mobile phase was a linear gradient elution of acetonitrile (A)–water (B) (v/v). The elution conditions were 0 min 19% A, 8 min 20% A, 12 min 36% A, 15 min 36% A, and 16 min 79% A. The current velocity was 0.22 ml min^−1^ and UV absorbance was measured at 203 nm.

### RNA isolation, RNA-seq data analysis and gene expression analysis

Total RNA of was extracted using Total RNA Purification Reagent (Tiangen, China). Subsequently, total RNA was qualified using agarose gel electrophoresis and quantified with a NanoDrop One Spectrophotometer and an Agilent 2100 Bioanalyzer (Thermo Fisher Scientific, USA). First-strand cDNA synthesis was performed with 5 μg of total RNA using GoScript™ Reverse Transcriptase (Promega, USA). Two RNA-seq libraries (main root and the rootlet) were generated and sequenced on the BGIseq500 platform by BGI-Shenzhen, China. The raw RNA-seq data have been submitted to NCBI with the accession numbers SAMN29967774 and SAMN29967775.

Gene expression analysis was performed by quantitative real-time PCR (qRT–PCR) on a CFX Connect™ Real-time System (Bio-Rad) as described previously [27]. The relative expression of genes was analyzed by the formula 2^-ΔΔCT^ and normalized to the expression of *P. notoginseng Actin2* (*PnACT2*). Gene expression was calculated based on three experimental replicates and three biological experiments. Gene-specific primers are indicated in Supplementary Data Table S1.

### Cloning of transcriptional regulators

The full-length cDNAs of *PnbHLH* [22] and *PnMYB1* [21] were amplified and identified as described previously. The full-length coding sequence of *PnMYB4* was amplified using the first-strand cDNA of *P. notoginseng* rootlets with primers designed according to the *P. notoginseng* transcriptomic dataset. After amplification, the amplicons were inserted into pLB-simple (Tiangen, Beijing) and transferred into Trans1-T1. Sanger sequencing was carried out by BGI-Beijing. The amino acid alignments and the phylogenetic tree of R2R3-MYB proteins were constructed with DNAMAN software and the MEGA 11.0 program, respectively, following previous reports [28].

### Subcellular localization of PnMYB4

*PnMYB4* was inserted into pCAMBIA1300-35S-GFP to yield a PnMYB4-GFP expression vector. The mesophyll protoplasts of *A thaliana* that were used for transient transfection assays were carefully isolated from leaves as described previously [29]. *A. thaliana* protoplasts were transiently transferred with 35S:PnMYB4-GFP, 35S:OsGhd7-RFP (nuclear marker) [30], or pCAMBIA1300-35S-GFP vector through a PEG-mediated method [29]. Localization of GFP and RFP was imaged with an FV10 laser scanning confocal microscope after 18–24 h transformation.

### Yeast one-hybrid assays

The open reading frame (ORF) sequence of *PnMYB4* was cloned into pGADT7 to yield pGADT7-PnMYB4 prey vectors. The promoter fragments upstream from the start codon of *PnSS* (1276 bp), *PnSE* (925 bp), and *PnDS* (855 bp) were amplified (Supplementary Data Table S2) and ligated to the pAbAi plasmid to yield pAbAi-PnSS-pro, pAbAi-PnSE-pro, and pAbAi-PnDS-pro bait plasmids, respectively. The recombinant bait constructs were digested and transferred into the yeast strain Y1H GOLD to yield bait-specific reporter strains. The prey vector was transferred into the bait yeast strains and then the transferred cells were cultured on SD plates lacking leucine with different concentrations of abscisic acid (AbA).

### Dual-luciferase assays

The complete ORFs of *PnMYB1*, *PnMYB4*, and *PnbHLH* were ligated to pGreenII 62-SK, while fragments of *PnSS*, *PnSE*, and *PnDS* promoters were amplified and subcloned into the pGreen II 0800-LUC to produce luciferase (LUC) reporters. These recombined plasmids were separately transformed into *Agrobacterium tumefaciens* EHA105 (pSoup-p19) and then infiltrated into leaf epidermal cells of *N. benthamiana*. After 2–3 days of incubation, the relative activity of LUC was analyzed with the Dual-Luciferase Reporter Assay Kit (Promega, USA).

### Electrophoretic mobility shift assays

The coding sequence of *PnMYB4* was subcloned into pET28a, and then the pET28a-PnMYB4 plasmid was translated into *Escherichia coli* BL21 cells. The recombinant PnMYB4 protein with His-Tag was purified using Ni-NTA HisTrap™ FF (Cytiva, Sweden). The probes (Supplementary Data Table S3) from the *PnSS, PnSE*, and *PnDS* promoters were synthesized and labeled with biotin with the Pierce Biotin 3′ End DNA Labeling Kit (Beyotime, Shanghai). Unlabeled probes with the same sequence as the biotin-labeled probes were used as competitors. Mutant probes with TTTTTT and GGGGGG, which were mutated from CAACC(T)A(G) and TAACCA, were synthesized and labeled, respectively. The remaining sequences were the same as those of the general probes. The electrophoretic mobility shift assay (EMSA) was carried out in 4% native acrylamide gel with an EMSA Kit (Beyotime, Shanghai) following a previous report [28].

### RNA interference of *PnMYB4*, *PnbHLH*, and *PnMYB1* expression in *P. notoginseng* calli

Fragments (200 bp) of *PnMYB3*, *PnMYB4*, *PnbHLH*, and *PnMYB1* were ligated into the pBWA(V)HS-RNAi vector. The resulting RNAi and empty vectors were transformed into *P. notoginseng* calli using an *A. tumefaciens* (GV3101)-mediated method described previously [31]. The calli were cultured on MS medium with 50 μg mL^−1^ hygromycin B and 300 μg mL^−1^ cefotaxime in the dark at 25°C for 8 days and then were collected for RNA extraction and saponin measurement.

### CRISPR/Cas9 of *PnMYB4* expression in *P. notoginseng* calli

One 23- to 24-bp guide RNA sequence target (ending with NGG) was synthesized and constructed into pKSE401 vector for precisely editing *PnMYB4* with the CRISPR/Cas9 system. The resulting CRISPR/Cas9 vector was transformed into *P. notoginseng* calli using the *A. tumefaciens* (GV3101)-mediated method as previously described [32]. The calli were cultured on MS medium with 50 μg ml^−1^ kanamycin and 300 μg ml^−1^ cefotaxime in the dark at 25°C and then were collected for DNA extraction, RNA extraction, and saponin measurement.

### Luciferase complementation imaging

The ORFs of *PnMYB4* and *PnMYB1* were amplified and cloned into pCAMBIA1300-cLUC, while the coding region of *PnbHLH* was introduced into pCAMBIA1300-nLUC. The resulting recombinant constructs were translated into *A. tumefaciens* EHA105 and then transfected into the leaves of *N. benthamiana*. The leaves were infiltrated with 1 mM d-luciferin and LUC activity was analyzed after 36–48 h using a multi-automatic chemiluminescence image analysis system (Tanon, China) [33]. For the competitive luciferase complementation imaging (LCI) assay, the ORFs of *PnMYB4* and *PnMYB1* were inserted into pCAMBIA1300-35S, and then the constructs were transformed into EHA105. The constructs were added to a mixture of PnMYB1/4-cLUC and PnbHLH-nLUC to demonstrate the competition between PnMYB1 and PnMYB4. The assay was performed as described above.

### Bimolecular fluorescence complementation assay

For the bimolecular fluorescence complementation (BiFC) assay, ORFs of *PnMYB4* and *PnMYB1* were cloned in frame with YFP^N^ into the pSPYNE-35S vector, while *PnbHLH* was cloned in frame with YFP^C^ into the pSPYCE-35S vector. The recombinant constructs were transferred into *Agrobacterium* strain GV3101. Agrobacteria containing constructs encoding the YFP^N^ and YFP^C^ fusion protein were co-transformed into *N. benthamiana* leaves as described previously [34]. After 48 h of incubation, YFP and chlorophyll autofluorescence were examined using an FV10 laser scanning confocal microscope and were observed at 514 and 633 nm, respectively.

### Co-immunoprecipitation assays

The coding sequences from *PnMYB4* and *PnMYB1* without the stop codon were ligated into pCAMBIA1300-35S-3xFlag, and *PnbHLH* was cloned into pCAMBIA1300-35S-Myc. *Nicotiana benthamiana* leaves were infiltrated with *A. tumefaciens* strain GV3101 carrying recombinant constructs as described previously [35]. After 2 days, the samples were harvested and ground in protein extraction buffer including 50 mM Tris–HCl (pH 7.5), 5% glycerol, 0.1% (v/v) Triton X-100, 0.5 mM phenylmethanesulfonyl fluoride (PMSF), 150 mM NaCl, protease inhibitor cocktail, 10 mM dithiothreitol (DTT), and 2% polyvinylpyrrolidone (PVPP), and total proteins were extracted at 100 rpm for 15 min at 4°C [36]. After centrifugation at 15 000 g at 4°C for 30 min, the supernatant was incubated with anti-Flag beads (Beyotime, China) at 4°C for 2 h. The beads were then washed four times using protein extraction buffer, and the immunoprecipitated protein complex was analyzed by western blotting using anti-Myc or anti-Flag antibodies.

### GST pull-down assays

The ORFs of *PnMYB4* and *PnMYB1* were cloned into pGEX-KG vector, which includes a glutathione-*S*-transferase (GST) tag. The PnMYB4 proteins with the GST tag were purified using the GST-Tag Purification Resin (Beyotime, China). Moreover, the ORF of *PnbHLH* was cloned into the pET28a vector, and the recombinant PnbHLH protein with His-Tag was purified by Ni-NTA HisTrap™ FF (Cytiva, Sweden). For the pull-down assay, 5 μg of purified His fusion PnbHLH, 5 μg of immobilized GST, GST-PnMYB3, GST-PnMYB4, or GST-PnMYB1 proteins and 50 μl GST-Tag Purification Resin were added to 1 mL GST pull-down binding buffer, and then incubated at 4°C overnight and centrifuged at 1000 g to remove the supernatant [37]. The remaining proteins were removed by GST pull-down binding buffer resuspension GST-Tag Purification Resin. Finally, SDS–PAGE loading buffer (2X) was put into the GST-Tag Purification Resin and boiled for 5 min for subsequent western blotting.

## Acknowledgements

This study was supported by Beijing Science and Technology Planning Project (Z201100005420005, China).

## Author contributions

S.W. and X.(Xiaohui)W. conceived and designed the research. J.M. and Y.S. conducted experiments and wrote the manuscript. Y.H. and X.Z. cloned the genes of PnMYB4 and analyzed the expression levels of the genes involved in saponin biosynthesis. X.(Xin)W. and S.L. analyzed the content of saponins. G.H. and K.A. performed the localization of PnMYB4. D.H. collected *Panax notoginseng* samples.

## Data availability

All relevant data and figures in this study can be found within the article and its supplementary materials. The raw RNA-seq data are available in NCBI and can be accessed with the accession numbers SAMN29967774 and SAMN29967775.

## Conflict of interest

The authors have no conflict of interest to declare.

## Supplementary data

Supplementary data is available at *Horticulture Research* online.

## Supplementary Material

Web_Material_uhad134
