# Supplementary material for: PnMYB4 negatively modulates saponin biosynthesis in Panax notoginseng through interplay with PnMYB1
Source: Hortic Res. 2023 Jul 5;10(8):uhad134. doi: 10.1093/hr/uhad134 (PMC10410195; doi:10.1093/hr/uhad134)
Supplement: Web_Material_uhad134 [file web_material_uhad134.zip › Fig S4-S7.pptx]

## Slide 1
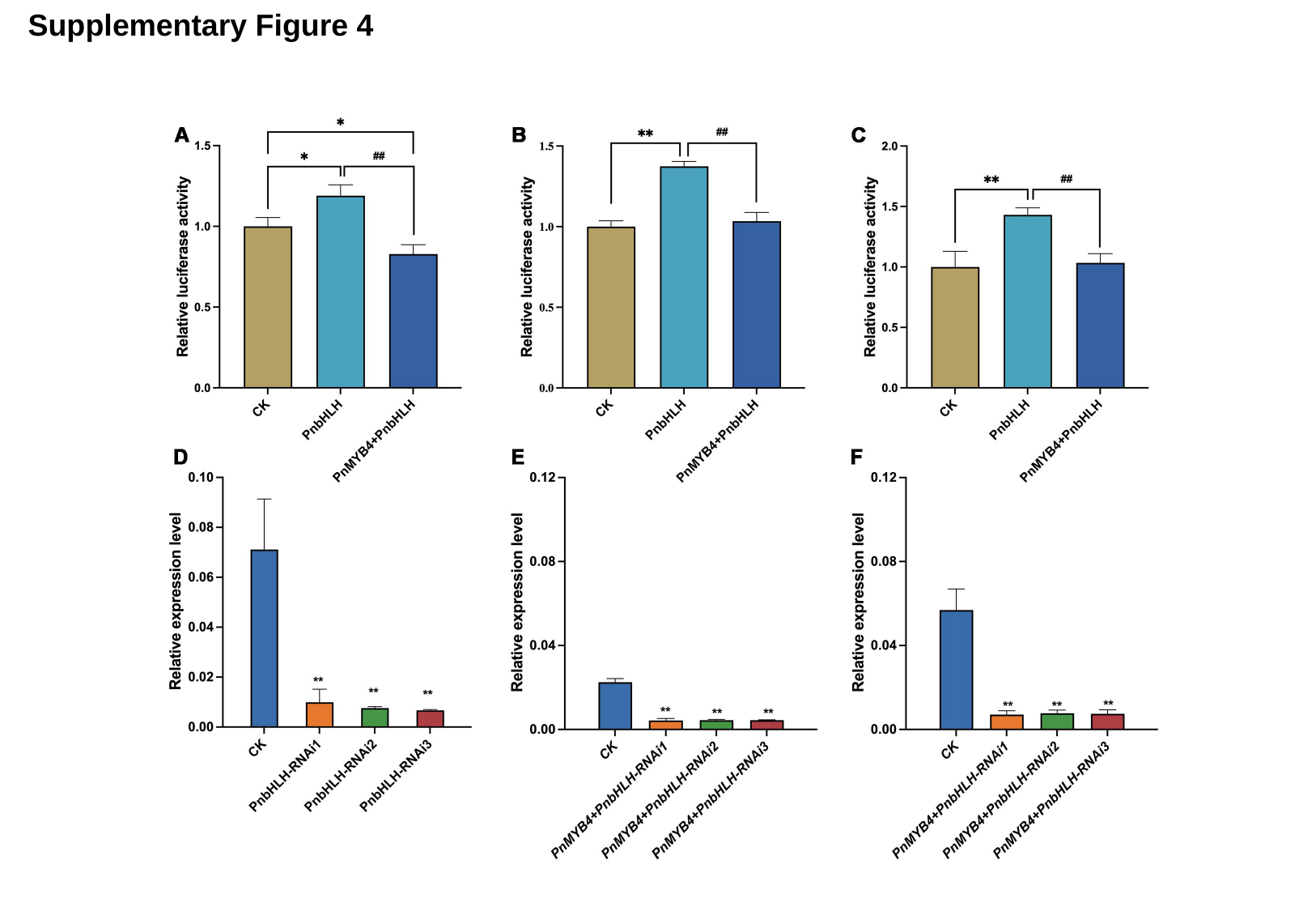

Supplementary Figure 4

## Slide 2
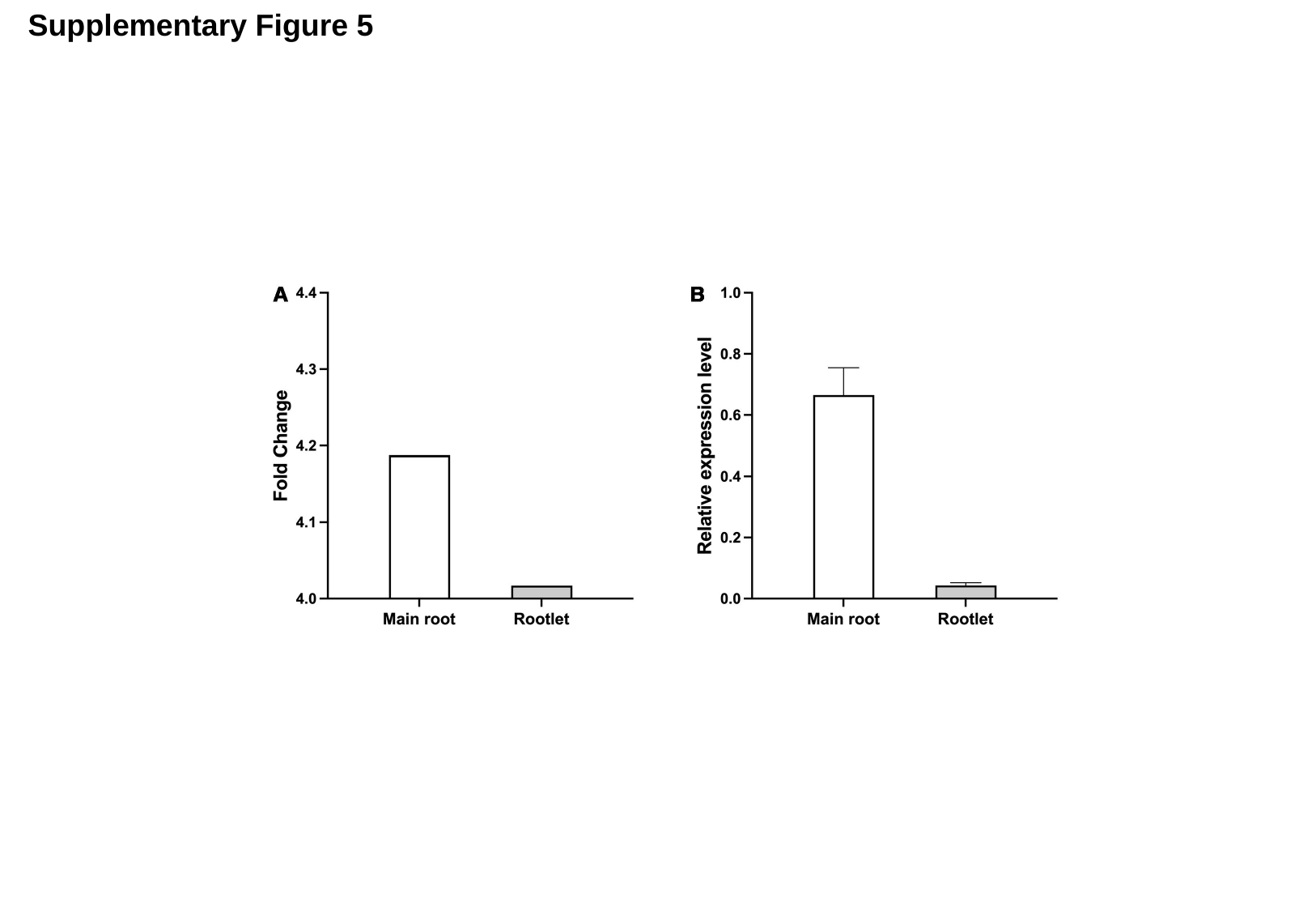

Supplementary Figure 5

## Slide 3
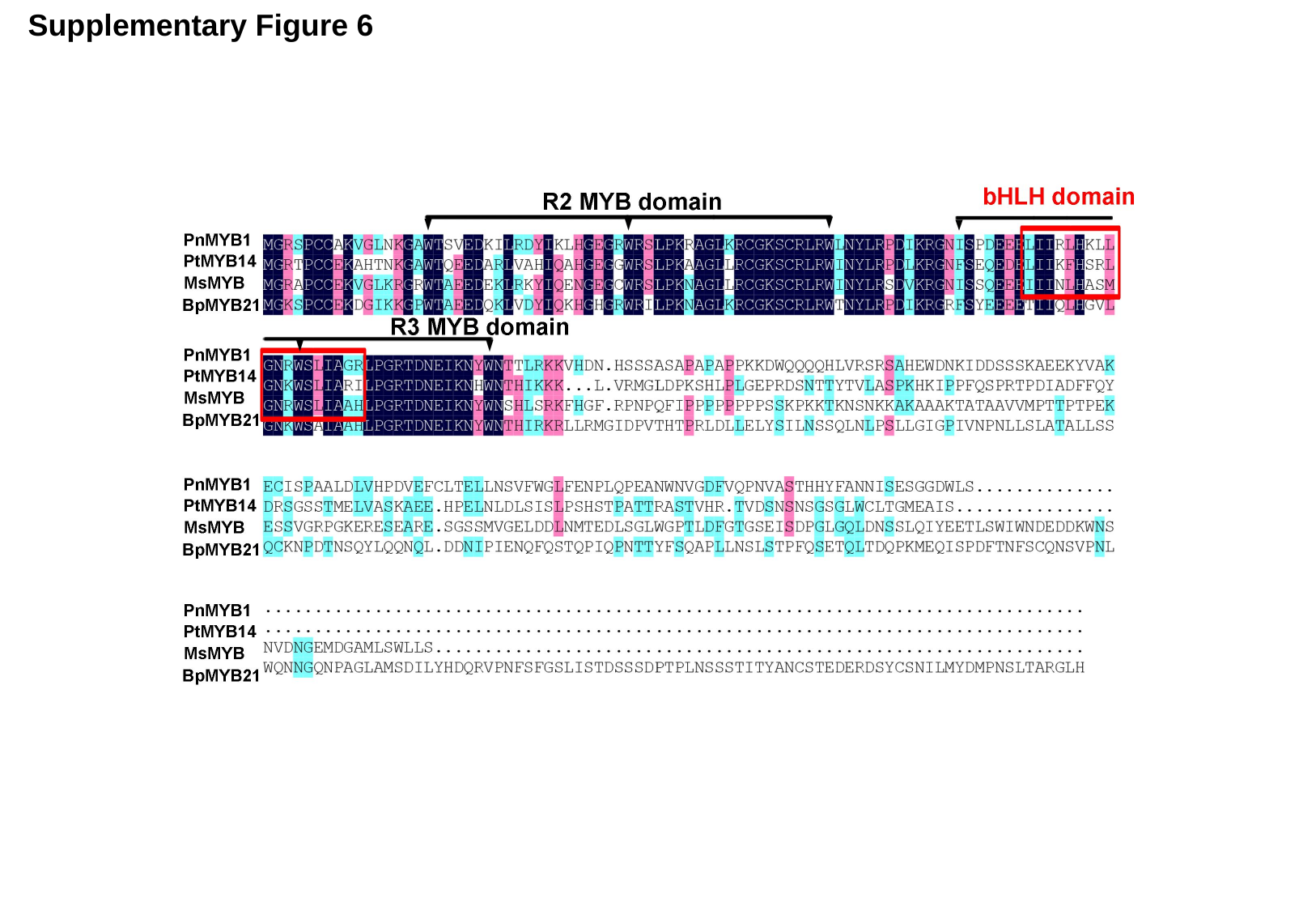

Supplementary Figure 6

## Slide 4
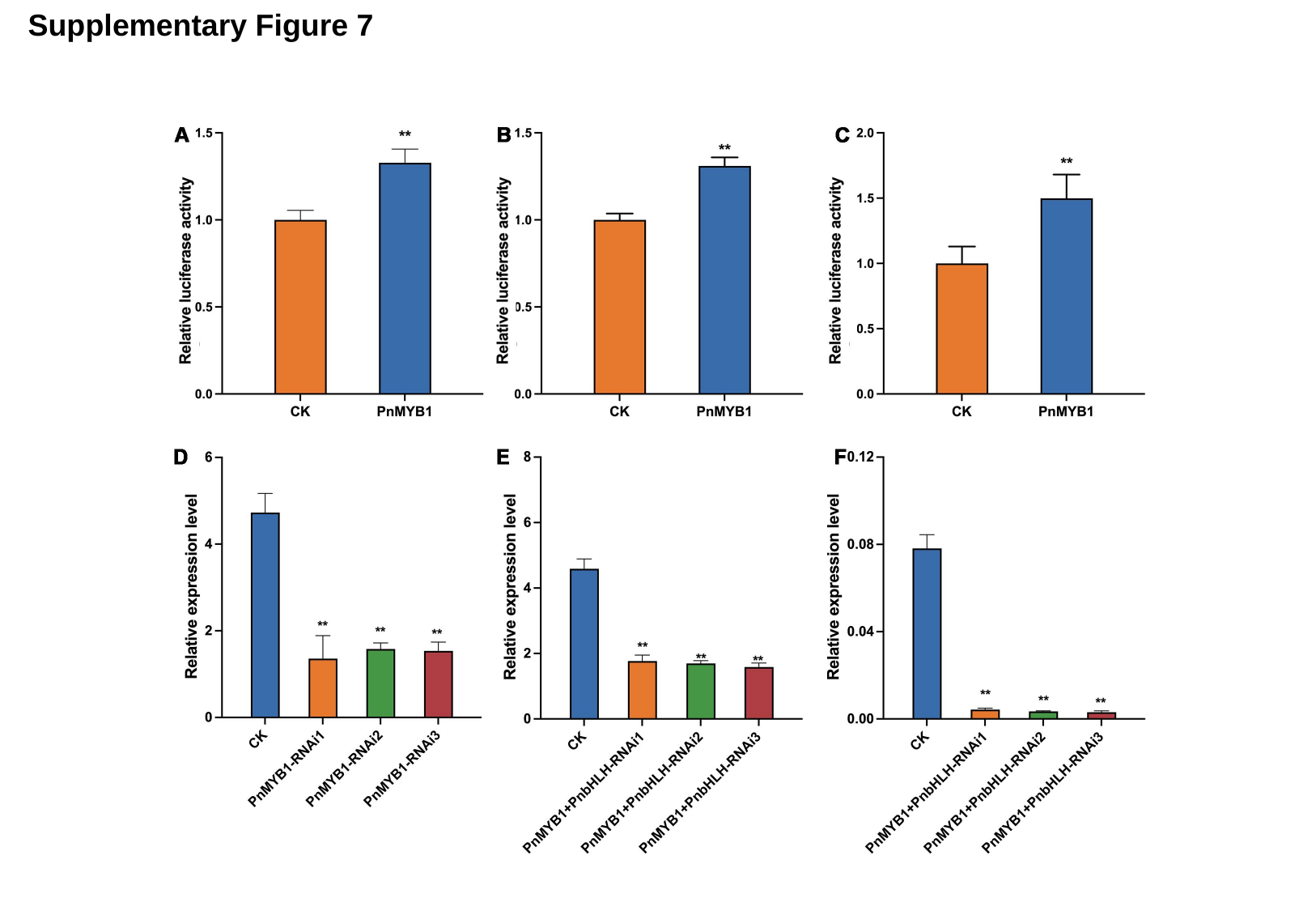

Supplementary Figure 7
