# Supplementary material for: PnMYB4 negatively modulates saponin biosynthesis in Panax notoginseng through interplay with PnMYB1
Source: Hortic Res. 2023 Jul 5;10(8):uhad134. doi: 10.1093/hr/uhad134 (PMC10410195; doi:10.1093/hr/uhad134)
Supplement: Web_Material_uhad134 [file web_material_uhad134.zip › Table S1.docx]

**TableS1** Primer sequences used for sequence amplification and qRT-PCR in *P. notoginseng*.

| Primer role | Primer name | Primers sequence (5'−3') |
| --- | --- | --- |
| Sequence  Amplification | PnMYB4-F1 | ATGGGAAGGTCCCCTTGTTGTGAGAAAG |
|  | PnMYB4-R1 | TCATTTCATCTCAATGCTTCTGTAG |
|  | PnMYB1-F1 | ATGGGGAGGAGCCCTTGCTGTGCAAAG |
|  | PnMYB1-R1 | TCAAGACAGCCAATCTCCTCCGGAC |
|  | PnbHLH-F1 | ATGGAGGATCCTTATTCCAATATCC |
|  | PnbHLH-R1 | TCACATTAACTGTTTGAGAGCTGCG |
|  | PnSS-pro-F1 | TACCGAGGGTTGATGAGAGGCATCT |
|  | PnSS-pro-R1 | CATTTTCTCTCTATATATCTGTTTCGC |
|  | PnSE-pro-F1 | GAGTGTTGCATATTTTTGAGTGCTT |
|  | PnSE-pro-R1 | GGTGTTGGTTGGACGTTCACGTTTC |
|  | PnDS-pro-F1 | CTTCCAATACTTGTAGTTTTGTGATT |
|  | PnDS-pro-R1 | CATTCTTCTACTACTTGTTGGTTATG |
| qRT-PCR | PnActin2-F | TCCAAGGGTGAATATGATGAATCG |
|  | PnActin2-R | AACCTCTCCAAAGAGAATTTCTGAGT |
|  | PnACAT-F1 | CTTTGAGCGTGGTATTGCTG |
|  | PnACAT-R1 | TCTCCTTGAAACTTGGTCGG |
|  | PnHMCAS-F1 | TGTGACAAGCAAGGACTGTAG |
|  | PnHMCAS-R1 | TGCCAAAGTTCCATTCTCGG |
|  | PnHMCAR/HMCGR-F1 | TGGTTAGGTTTACTACGGCG |
|  | PnHMCAR/HMCGR-R1 | TCCCTGCCATTGAGCATTG |
|  | PnMVK-F1 | AATCAAGGGTTGCTCCAGTG |
|  | PnMVK-R1 | TGTGGGTAACAGTGTCAGAAC |
|  | PnPMK-F1 | CGGCTGTTGGATGCTACTATG |
|  | PnPMK-R1 | ACTGCTCTGTAAAGAAACGCC |
|  | PnMDD-F1 | TTACGGCTCAAACGCCAAC |
|  | PnMDD-R1 | GGACTCACCGAAACAGTAGTAG |
|  | PnDXS-F1 | CTTTGAGCGTGGTATTGCTG |
|  | PnDXS-R1 | TCTCCTTGAAACTTGGTCGG |
|  | PnDXR-F1 | CACTATTAGGCAAACTTCAGGG |
|  | PnDXR-R1 | ATACCAAGACCAGCAGAGATG |
|  | PnispD-F1 | TCTACACTTTCTCACTCTTGCC |
|  | PnispD-R1 | AATCTTGTCTCTCCTTTCCAGG |
|  | PnispE-F1 | ACCTCTACAACTCCAATAGCC |
|  | PnispE-R1 | GAACCAGAAGCCATTACTGTG |
|  | PnispF-F1 | GCTGGGAAACTTAGATGCGAC |
|  | PnispF-R1 | TCAGGTTCACAACGGAAGGG |
|  | PnispH-F1 | GGTGATGTAGTGATTCTGCCTG |
|  | PnispH-R1 | AGCAACAGTCTCCTCGTGAG |
|  | PnispG-F1 | GATGGACGGATGAAGTCTGC |
|  | PnispG-R1 | CATACCAAGGTTAGCCAGCC |
|  | PnIDI-F1 | AACTGGGTATTCCTGCTGAAG |
|  | PnIDI-R1 | TCCCGAACAATGAAGAGAAGG |
|  | PnGGPS-F1 | GCCGTTTGGGTTTGAACTAC |
|  | PnGGPS-R1 | CTCGCCTTCCTTCTTCTCAC |
|  | PnFPDS-F1 | ACTCAACGACCCTGCTTTC |
|  | PnFPDS-R1 | TCAATAACAGACAGCCCTCG |
|  | PnCAS-F1 | ATCCAGGCAGTGTAGTCTTAC |
|  | PnCAS-R1 | CAGGCATTAGAAACATAGGACC |
|  | PnSS-F1 | CTATGTAGCAGGACTTGTTGG |
|  | PnSS-R1 | ATGCGTGACTTTGGTATCTC |
|  | PnSE-F1 | TTTGATTACCTGAGCCTCG |
|  | PnSE-R1 | CCAACGCCATAAATAGCC |
|  | PnDS-F1 | AGATAGAGGATTCTGTGGCG |
|  | PnDS-R1 | CGAACTGCTTCACTGTTGTC |
|  | PnMYB4-F2 | ATCAGCACCAAACAGAGGC |
|  | PnMYB4-R2 | CAACCCTAAGAAATCATAGCCG |
|  | PnMYB1-F2 | GCAGGTTTAAAGATATGTGGGAAG |
|  | PnMYB1-R2 | CTGAAGCAGAGGAGGAGTGATTG |
|  | PnbHLH-F2 | AAGATGGACAAGGCTTCAG |
|  | PnbHLH-R2 | TCACAAAGACCACAGATTCC |
|  | CL1605.Contig9_All-F1 | GGGACTGGAGGAACATTTCAC |
|  | CL1605.Contig9_All-R1 | CTGTCAGATGGACCGTTGTG |
|  | CL1806.Contig2_All-F1 | TCAAGTGATGATGCCACGG |
|  | CL1806.Contig2_All-R1 | AGGTCAGAAAGCGTAGACAC |
|  | CL1806.Contig5_All-F1 | GCCAGGAAGAACTGATAATGAG |
|  | CL1806.Contig5_All-R1 | AATCTCGGCATCCAAAGG |
|  | CL3810.Contig1_All-F1 | ACGACCTGAAATAGACCTGTC |
|  | CL3810.Contig1_All-R1 | GGATACTGGTGGAATGATAGTG |
|  | CL2694.Contig1_All-F1 | CTCGCTTACCAGGAAGAACC |
|  | CL2694.Contig1_All-R1 | GAGATGATGAGTTGTGAAAGGC |
|  | CL8936.Contig1_All-F1 | ATTGCTGCTCAGTTGCCTG |
|  | CL8936.Contig1_All-R1 | CCTTCTCCACATTTAGGTCTCC |
|  | Unigene23157_All-F1 | GTAACAGGTGGTCTAAGATTGC |
|  | Unigene23157_All-R1 | ATGCTCAAGAGGTTTCTGGG |
|  | Unigene59629_All-F1 | CTATCCTTGGCAATAAGTGGTC |
|  | Unigene59629_All-R1 | AGAGCAATAAGATGAGGCAGG |
| Subcellular localization | PnMYB4-F3 | CGGGGTACCATGGGAAGGTCCCCT |
|  | PnMYB4-R3 | TGCTCTAGATTTCATCTCAATGCT |
| Yeast One-Hybrid Assays | PnMYB4-F4 | AGTGAATTCATGGGAAGGTCCCCT |
|  | PnMYB4-R4 | CAGCTCGAGTTATTTCATCTCAATG |
|  | PnSS-pro-F2 | CGGGGTACCCGGTACGCG  CGGATCTTCCAGAGATTTAC |
|  | PnSS-pro-R2 | ACGCGTCGACTTTGCA  CGCCTGCCGTTCGACG |
|  | PnSE-pro-F2 | CGGGGTACCCGACTTGGA  CATGATTACGCCAGTTTG |
|  | PnSE-pro-R2 | ACGCGTCGACTGGGGTAA  CGACGGCAGTGATTAGAAC |
|  | PnDS-pro-F2 | CGGGGTACCCTCGGTAC  GCGCGGATCTTCCAGAG |
|  | PnDS-pro-R2 | ACGCGTCGACCGCC  TGCCGTTCATTCTTC |
| Dual luciferase transcriptional activity assay | PnMYB4-F5 | CGCTCTAGAACTAGTGGATCC  ATGGGAAGGTCCCCTTGTTG |
|  | PnMYB4-R5 | TTGATATCGAATTCCTGCAG  TTATTTCATCTCAATGCTTC |
|  | PnMYB1-F3 | GGAGAGGACAGCCCAAGCTGAGCTC  ATGGGGAGGAGCCCTTGCTGTG |
|  | PnMYB1-R3 | GCCCGGGGGATCCACTAGTTCTAGA  TCAAGACAGCCAATCTCCTCCGG |
|  | PnbHLH-F3 | CGCTCTAGAACTAGTGGATCCAT  GGAGGATCCTTATTCCAATATCC |
|  | PnbHLH-R3 | TTGATATCGAATTCCTGCAG  CTACATTAACTGTTTGAGAGC |
|  | PnSS-pro-F3 | ACGCGTCGACCGGTACGCG  CGGATCTTCCAGAGATTTAC |
|  | PnSS-pro-R3 | CGCGGATCCTTTGCAC  GCCTGCCGTTCGACG |
|  | PnSE-pro-F3 | ACGCGTCGACCGACTTGG  ACATGATTACGCCAGTTTG |
|  | PnSE-pro-R3 | CGCGGATCCTGGGGTAAC  GACGGCAGTGATTAGAAC |
|  | PnDS-pro-F3 | ACGCGTCGACCTCGGTAC  GCGCGGATCTTCCAGAG |
|  | PnDS-pro-R3 | CGCGGATCCCGCCTGCCG  TTCGACGATTCATTCTTC |
| Electrophoretic mobility shift assay | PnMYB4-F6 | CGCGGATCCATGGGAAGG  TCCCCTTGTTGTGAGAAAG |
|  | PnMYB4-R6 | ACGCGTCGACTCATTTCA  TCTCAATGCTTCTGTAG |
| BIFC | PnMYB4-F7 | GGCGCGCCACTAGTGGATCC  ATGGGAAGGTCCCCTTGTTG |
|  | PnMYB4-R7 | GCGGTACCCTCGAGGTCGAC  TTTCATCTCAATGCTTCTGTAG |
|  | PnMYB1-F4 | CTGGCGCGCCACTAGTGGATCC  ATGGGGAGGAGCCCTTGC |
|  | PnMYB1-R4 | GAGCGGTACCCTCGAGGTCGAC  AGACAGCCAATCTCCTCCGG |
|  | PnbHLH-F4 | GGCGCGCCACTAGTGGATCC  ATGGAGGATCCTTATTCCAA |
|  | PnbHLH-R4 | GCGGTACCCTCGAGGTCGAC  CATTAACTGTTTGAGAGC |
| COIP | PnMYB4-F8 | ACGAGCTCGGTACCCGGGGATCC  ATGGGAAGGTCCCCTTGTT |
|  | PnMYB4-R8 | CATGGTCTTTGTAGTCCATGTCGAC  TTTCATCTCAATGCTTCTG |
|  | PnMYB1-F5 | ACGAGCTCGGTACCCGGGGATCC  ATGGGGAGGAGCCCTTGCTGTGC |
|  | PnMYB1-R5 | CATGGTCTTTGTAGTCCATGTCGAC  AGACAGCCAATCTCCTCCGGAC |
|  | PnbHLH-F5 | ACACGGGGGACGAGCTCGGTACC  ATGGAGGATCCTTATTCCAA |
|  | PnbHLH-R5 | TCCATGTCGACTCTAGAGGATCC  CATTAACTGTTTGAGAGC |
| GST PULL-DOWN | PnMYB4-F9 | GGGAATTTCCGGTGGTGGTGGTG  GAATTCTAATGGGAAGGTCCCCTTGTTG |
|  | PnMYB4-R9 | CACGATGAATAAGCTTGAGCTCGAG  TTATTTCATCTCAATGCTTCTGTAGTCC |
|  | PnMYB1-F6 | GGGAATTTCCGGTGGTGGTGGTG  GAATTCTAATGGGGAGGAGCCCTTGC |
|  | PnMYB1-R6 | CACGATGAATAAGCTTGAGCTCGAG  TCAAGACAGCCAATCTCCTCCGG |
|  | PnBHLH-F6 | TGGGTCGCGGATCCGAATTC  ATGGAGGATCCTTATTCCAA |
|  | PnBHLH-R6 | GTGCTCGAGTGCGGCCGCAAGCTT  CATTAACTGTTTGAGAGCTGCG |
| LCI | PnMYB4-F10 | ATCTCGTACGCGTCCCGGGGC  GGTACCATGGGAAGGTCCCCTTGTT |
|  | PnMYB4-R10 | AGTGGCGCGCCGGGCCCTCTAGA  TTATTTCATCTCAATGCTTCTGTAGTCC |
|  | PnMYB1-F7 | ATCTCGTACGCGTCCCGGGGC  GGTACCATGGGGAGGAGCCCTTGC |
|  | PnMYB1-R7 | AGTGGCGCGCCGGGCCCTCTAGA  TCAAGACAGCCAATCTCCTCC |
|  | PnbHLH-F7 | AGAACACGGGGGACGAGCTCGGTACC  ATGGAGGATCCTTATTCCAATATCC |
|  | PnbHLH-R7 | ACGCGTACGAGATCTGGTCGAC  CATTAACTGTTTGAGAGCTGC |
|  | PnMYB4-F11 | CGGGGTACCATGGGAAGGTCCCCT |
|  | PnMYB4-R11 | CGCGGATCCTTATTTCATCTCAAT |
