# Supplementary material for: PnMYB4 negatively modulates saponin biosynthesis in Panax notoginseng through interplay with PnMYB1
Source: Hortic Res. 2023 Jul 5;10(8):uhad134. doi: 10.1093/hr/uhad134 (PMC10410195; doi:10.1093/hr/uhad134)
Supplement: Web_Material_uhad134 [file web_material_uhad134.zip › Table S2.docx]

**TableS2** The promoter sequence of *PnSS*, *PnSE* and *PnDS*.

(yellow means probed sequence, red means MYB binding sites)

| Promoter name | Promoter sequence (5'−3') |
| --- | --- |
| *PnSS-promoter* | ACCGAGGGTTGATGAGAGGCATCTAGGAGTCCTCGCTGGAAAGAAACACTGTTAATTAATGTGATGAGAAGACAGTGTTTGGGCTCCTAAAATAAAATATGAGTTCTTACAGGAAAACGGGACATGAAATGTATTTAAATTTTAATTATTTATATTTTTATTCTTTTTAGATGTCAAAATACAAATATGTGACCTAATTTTCTGTCCCATTTTCTTGTTCACCAAAAGTTTTTNATAAAATATTCATTATTAACTTAGTGGTCAAATACTAATAATTAAAGATGGAAAATAAAATAATAAAAAAAGGAATTTGTCTCAAATAAATGAGAGTCATGCATATGTTTTCTTTATTTCTTTTATTTTATTTTCTTTTTTCAAAGGTCACGTTAAATTTTTAAAAAATGAATGAACTTTTTAAAATAAAATTATCTTTTAAAACGTACGAAATAACACTTAACAACTCGATAAATATACGATGAACAAACTTGATAAATAAATAATCATGAATATTAATGTTGAATTCGCTAGATATACTAATTGTAGTAACATTTTGTCATAAGCAATCTTGTATCTCTAGTCTCTAATTAATGTTTATCTTGACTTAATAAATAAAATTTATAAATAACAAAGTTTTTGGGATTAAATTTTAGTGTAAAAAAATAAAATAACAAAATTATTTTAACAACCAACTTATATTTTAATTTATAAATTATTTAAAAATAAATCATTCCCGAACTCCCATCTTCTAACGAGCAACTTGAGTTCCGTATTTACTTATTAGCTTTTTCTCGTACTTCCGATATTATTTTGAACTACTTAGATAATTTATTAAAATGAGTAAAAAAAATAAAAATCACTTGTATTTTATGAAATAAATATTCAAAATAAATAATTATTTCCTCGCCGTTTCTTTTCTCTCCGCTGTCTCCGTCTCTCTGTCGTATTCGTTTTGCATCTTTATCGAATCCGTGAGTCTCTGTCTTTCAATTTCCGTCTTCATTTTGACAAATCAAAATTCCTCCCAAACAGAAATCACTAATTTTATCCCCTCAAAATATATGGAAGGAGTACTAAACTCGTCCAAACAATAATTCACTATTCCATTTTCATTTCCATTTCACATTCCAACTGCAAATTAAGCAAGCTTATTATAAAGTGAGCAAATTCGTTTGAAAAATCTCAATGTTCTTTATAATCATTCAATTAATTGCTTTAGATCTGTTCATTTCATTCATTTTTTTTGTGCTTGGCGAAACAGATATATAGAGAGAAAATG |
| *PnSE- promoter* | TTTATCGTCAAAATCTTATCAACTACTACTACATTCTTATGGGGCAGATATATTTTTTTCATAAATATGGGGGTTGGTAGATTTATATCCCTTTTTTTTTATAATTATAGGGAAAATAATCAAATGTTAAAAATAAAGAATATTTACACAGACATGGTCATGGGATGCTAGAATAGTTATAAATGAGGGGAGAGAAACAAAAAATAATAGTAATAGTGCGGACAGAAAATTAGGTGTTAAATGAGTGAGAACGTATAAAATTAAGAATAATGTTACTATCATATAAAAAAAGTTTTATGTATTTACATACATACACATGCATATTGTGTGCTCCGCCTGATATGTGGGGCCCGCGGTTATGCATGTTTGTATATGTGAGTATTATGTGACCATTTTTATGTGATAGTAGCATTATTTTAAAATTAATTACAAACATAAATATTGGTTGATGTATGTGCCTCCAATTAAATGTATAAATTAGGTGTATAATATTAGATGAGCTGAATGACACTGCTGTGTATCTAAGTTGTTATATAGTTTTAAGATCCTCTTTAAAATTTATTTTTCGTTAATTAAATCTTTAAAGTGACATTTATGTCAAATTCGTTCATTTTTCCCGATTTGACCTTAATTTCCCTACAAAGAAAGTAATATGATAACCATAAATAAATAAAATATTAAAACTTAAAAACAAGTCTATCTATTCTTCCTACTTGAAGGGTAAAAAACCAAACCTATTCATTTGCTCCGCGCAACACCACTCCATTTCCATCTGATCTGACCCACAAAAAGAAATCTCAAAATCCATTTCAATTTGTGGTTTCAATTTCAATTGCAGTGGAGGTGTATATACAATAATCAGCTTCTGCCAATTGTTGTTGAATATATGGATTGAAATGAAAGCACTCAAAAATATGCAACACTC |
| *PnDS-promoter* | CCAATACTTGTAGTTTTGTGATTTTCCAAATTTGACAGCTAGTAGTAGAACTAGGTGGTTAAACTAGAAATCAATAATTTTTCTTAACTGCACAATCTGCATAACAATAAGCCCTACAAGAAGCAATAGTAAATCATTATAGTACTGATTTACTACTACTCTCAATTTTATCTTTTTTAGGACAAATAAAATAATTATCATAATCAAATCCCACAAAATGGAATAATAAAAAAAAAGAAACATTAAAACTTATTTGAAGCTTATAAAGATACCAAAACTGGTTTATACTTTGAAATTAATTCATGAGTTTGTTTGAATATAGAAAATAAATTTGGATCGAGGCTTTAAGCATTTTGGGAATCATTCAAATTGAATTATGACAAATAATCGAAGTAAGTTTCTAATTGTATCTAAATATTTTTTATTGGAAATTTCTTTTTTAGTTTAGATTATGAATATTTATTGCAATTTGTAAACATTCTTATGATTAGTTTAAATTTTTTTTATTTAAGTTTAAATAAAGAAAAATTCCTAGATCCGCCCCTGATAAAGAGTACAATTATATTTTTCTAGAAGGAATAATTTTTTAATAATGCAGGTGTATAATTCTACAAAAAAGGAGTATAATTTTTTTTTTAAAAAAGGATGGGCACATTTTTAACGTTGTACGTTATTTCTTCCATGTTTATATCGTGCGTAAATATACAAGTGGACTTTATGTCTAGATCTAGAGTGGCTAGATGTTTATATATTATCAGCTCCACAACATTTGGGTGGTATATAATAAATAATGGTTCAACTACAATAAATATAATTAATATATATATATAATAGATAATTAAGCCATCTATGTACACCACATAACCAACAAGTAGTAGAAGAATG |
