# Supplementary material for: PnMYB4 negatively modulates saponin biosynthesis in Panax notoginseng through interplay with PnMYB1
Source: Hortic Res. 2023 Jul 5;10(8):uhad134. doi: 10.1093/hr/uhad134 (PMC10410195; doi:10.1093/hr/uhad134)
Supplement: Web_Material_uhad134 [file web_material_uhad134.zip › Table S3.docx]

**TableS3** Probe sequences used for Electrophoretic mobility shift assay.

| Sequence role | Probe name | Probe sequence (5'−3') |
| --- | --- | --- |
| Electrophoretic mobility shift assay | PnSS-pro-probe-F1 | ATTATTTTAACAACCAACTTATATTT |
|  | PnSS-pro-probe-R1 | AAATATAAGTTGGTTGTTAAAATAAT |
|  | PnSS-pro-probe-mutant-F1 | ATTATTTTAATTTTTTACTTATATTT |
|  | PnSS-pro-probe- mutant-R1 | AAATATAAGTAAAAAATTAAAATAAT |
|  | PnSS-pro-probe-F2 | TTTCACATTCCAACTGCAAATTAAGC |
|  | PnSS-pro-probe-R2 | GCTTAATTTGCAGTTGGAATGTGAAA |
|  | PnSS-pro-probe-mutant-F2 | TTTCACATTCTTTTTTCAAATTAAGC |
|  | PnSS-pro-probe-mutant-R2 | GCTTAATTTGAAAAAAGAATGTGAAA |
|  | PnSE-pro-probe-F1 | ACATAAATATTGGTTGATGTATGTGC |
|  | PnSE-pro-probe-R1 | GCACATACATCAACCAATATTTATGT |
|  | PnSE-pro-probe-mutant-F1 | ACATAAATATAAAAAAATGTATGTGC |
|  | PnSE-pro-probe-mutant-R1 | GCACATACATTTTTTTTATATTTATGT |
|  | PnSE-pro-probe-F2 | AGTAATATGATAACCATAAATAAATA |
|  | PnSE-pro-probe-R2 | TATTTATTTATGGTTATCATATTACT |
|  | PnSE-pro-probe-mutant-F2 | AGTAATATGAGGGGGGTAAATAAATA |
|  | PnSE-pro-probe-mutant-R2 | TATTTATTTACCCCCCTCATATTACT |
|  | PnDS-pro-probe-F1 | TAGAACTAGGTGGTTAAACTAGAAAT |
|  | PnDS-pro-probe-R1 | ATTTCTAGTTTAACCACCTAGTTCTA |
|  | PnDS-pro-probe-mutant-F1 | TAGAACTAGGCCCCCCAACTAGAAAT |
|  | PnDS-pro-probe-mutant-R1 | ATTTCTAGTTGGGGGGCCTAGTTCTA |
|  | PnDS-pro-probe-F1 | GTACACCACATAACCAACAAGTAGTA |
|  | PnDS-pro-probe-R1 | TACTACTTGTTGGTTATGTGGTGTAC |
|  | PnDS-pro-probe-mutant-F1 | GTACACCACAGGGGGGACAAGTAGTA |
|  | PnDS-pro-probe-mutant-R1 | TACTACTTGTCCCCCCTGTGGTGTAC |
