# Supplementary figures and images for: PnMYB4 negatively modulates saponin biosynthesis in Panax notoginseng through interplay with PnMYB1
Source: Hortic Res. 2023 Jul 5;10(8):uhad134. doi: 10.1093/hr/uhad134 (PMC10410195; doi:10.1093/hr/uhad134)

## Slide 1
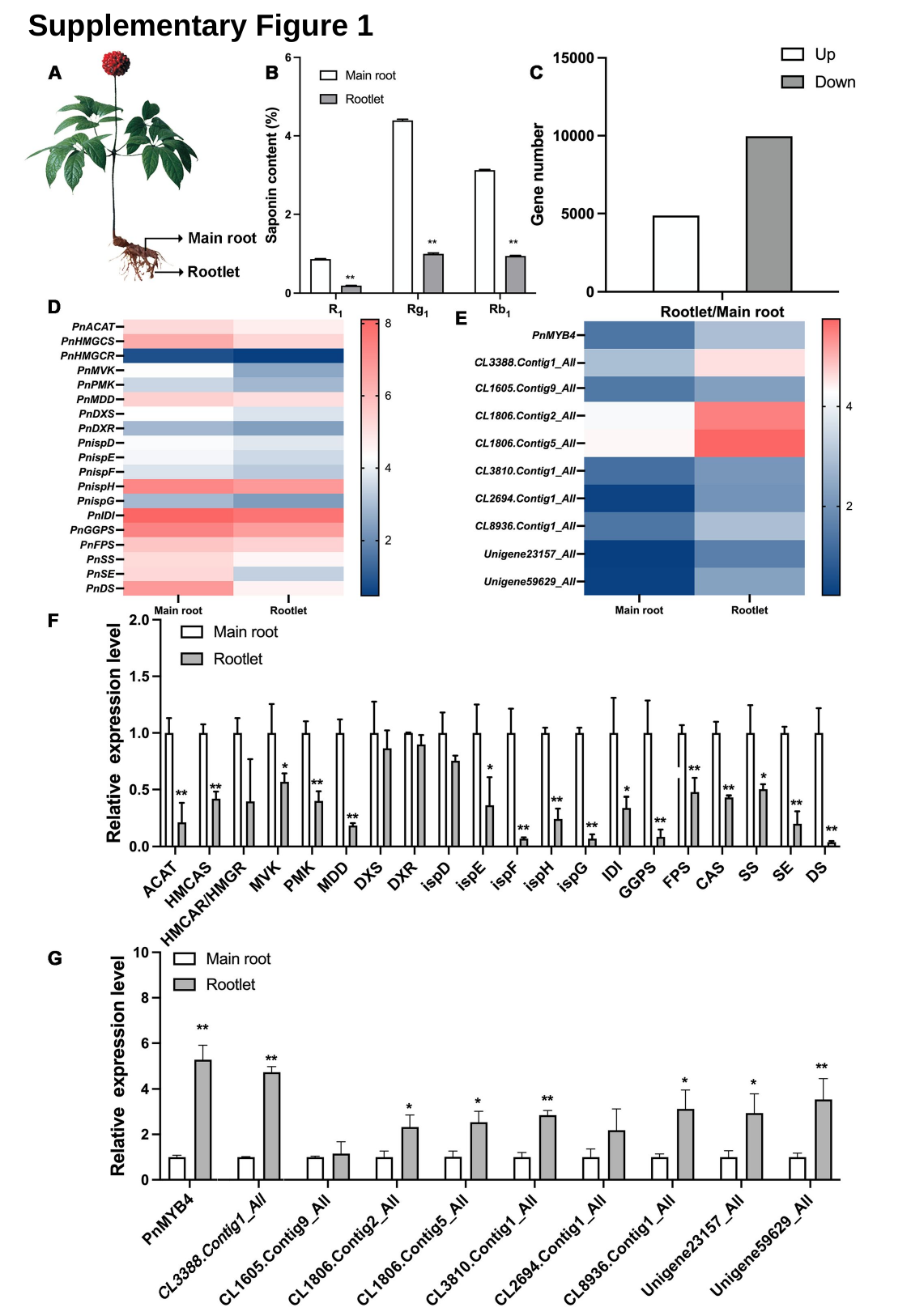

Supplementary Figure 1

Supplement: Web_Material_uhad134 [file web_material_uhad134.zip › Fig S1.pptx]

## Slide 1
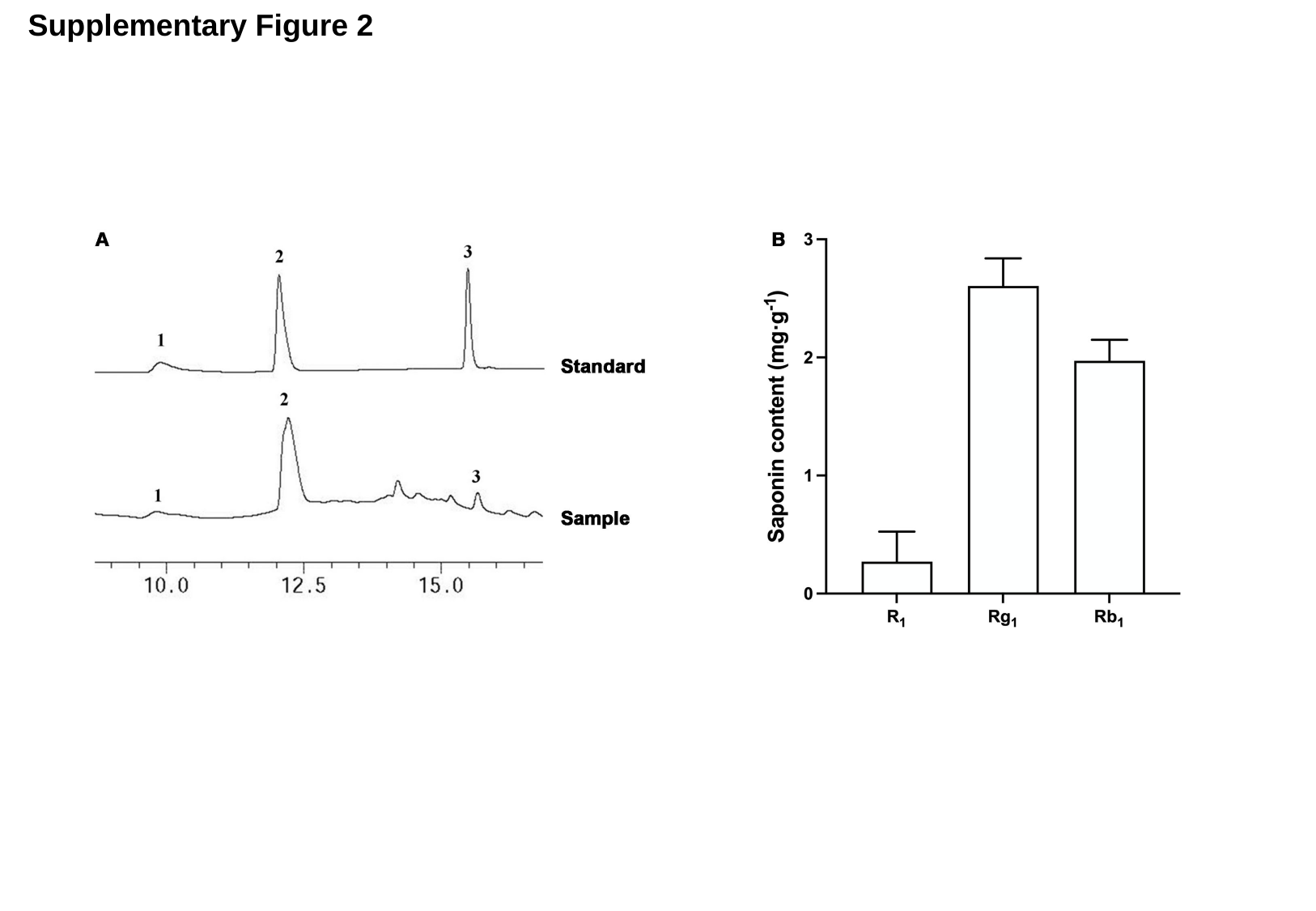

Supplementary Figure 2

Supplement: Web_Material_uhad134 [file web_material_uhad134.zip › Fig S2.pptx]

## Slide 1
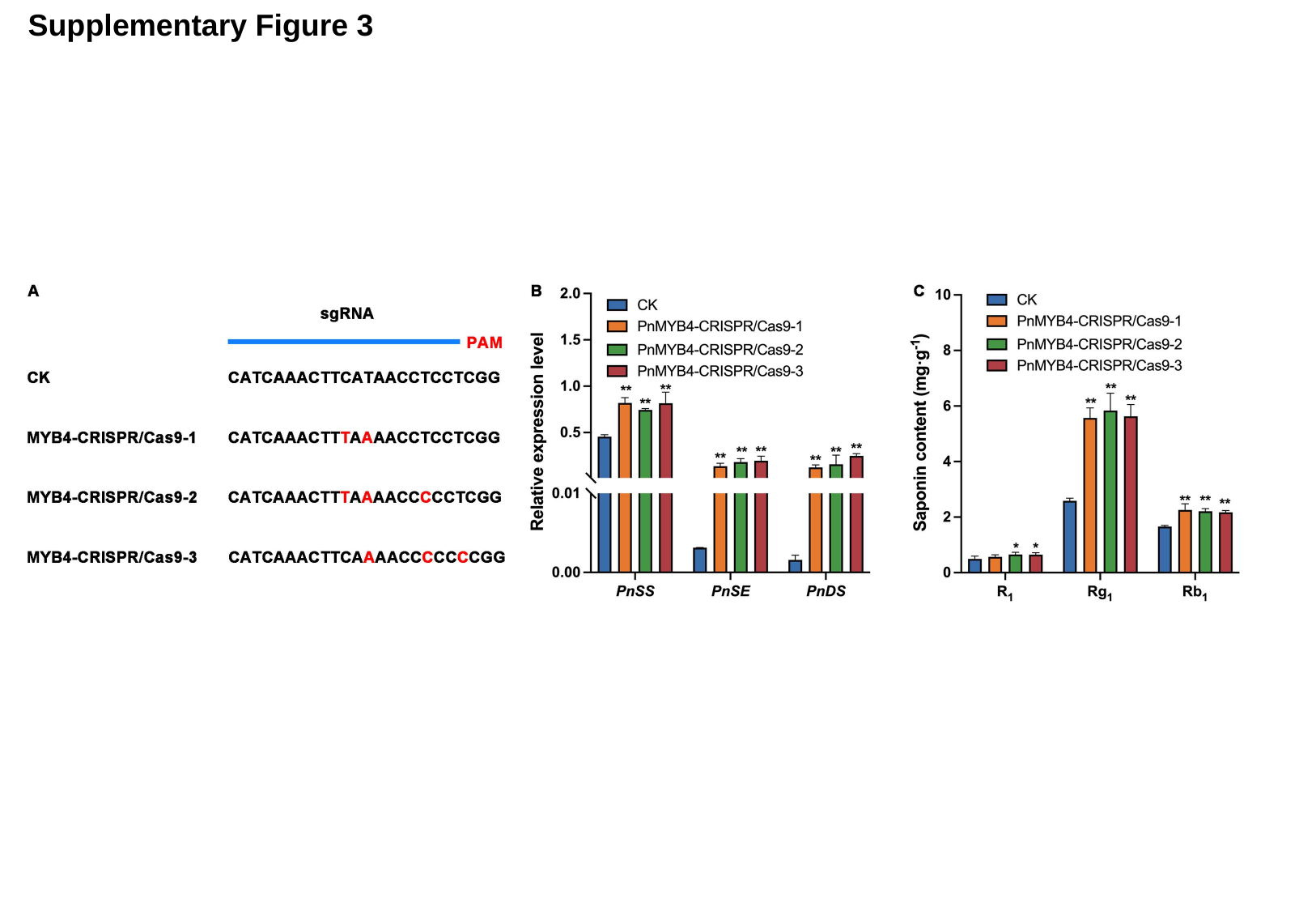

Supplementary Figure 3

Supplement: Web_Material_uhad134 [file web_material_uhad134.zip › Fig S3.pptx]
